# Effects of Digital Nature Interventions on Positive Psychological Outcomes: A Scoping Review

**DOI:** 10.3390/bs16071257

**Published:** 2026-07-22

**Authors:** Dev Bandhu Poudel, Karl E. Mitchell, Samjhana Acharya, Chandra Gurung, Yasuhiro Kotera

**Affiliations:** 1 Department of Humanities and Social Sciences, G. P. Koirala Memorial (Community) College, Kathmandu 44600, Nepal; 2 Department of Humanities and Social Sciences, Brooklyn College, Kathmandu 44600, Nepal; derajung@gmail.com; 3 Research Department, Sambhavya Foundation, Kathmandu 44600, Nepal; 4 School of Psychology, University of Derby, Derby DE22 1GB, UK; k.e.mitchell@derby.ac.uk; 5 Central Department of Rural Development, Tribhuvan University, Kathmandu 44600, Nepal; samjhoo44@gmail.com; 6 Faculty of Medicine and Health Sciences, University of Nottingham, Nottingham NG7 2UH, UK; 7 Center for Infectious Diseases Research and Education, The University of Osaka, Suita 565-0871, Japan; 8 Department of Social Sciences, Azerbaijan University, Baku AZ1007, Azerbaijan

**Keywords:** digital nature, virtual reality, positive psychology, well-being, stress reduction, nature connectedness, restorativeness, immersive technology, mental health, scoping review

## Abstract

(1) Background: Digital nature, delivered through different media, has been proposed as a scalable substitute for physical nature exposure to support psychological well-being, especially in high-income countries. This scoping review aimed to map the evidence on digital nature interventions and positive psychological outcomes. (2) Methods: Following PRISMA-ScR guidelines, we searched PubMed, ScienceDirect, and Google Scholar, supplemented by AI search and citation tracking, for studies published between 2020 and 2025. The first search was conducted between 15 November 2025 and 25 December 2025 and revisited between 5 June 2026 and 10 June 2026. We included articles published in English in peer-reviewed journals with the full text available. (3) Results: Our search yielded 67 studies from diverse cultural settings in three categories: primary studies (*N* = 46), review articles (*N* = 19) and conceptual articles (*N* = 2). Digital nature was associated with reduced stress and anxiety, better mood, greater restorativeness, and stronger nature connectedness, across healthy individuals, older adults, and clinical populations; reviews supported these findings at a larger scale, though some flagged mixed results for specific outcomes and technologies. Two conceptual articles explained these patterns through biophilic design and digital placemaking. (4) Conclusion: Overall, digital nature appears to be a promising approach for promoting psychological well-being, particularly where access to natural environments is limited. However, the evidence is mixed, geographically concentrated in Europe, East Asia, and North America, and methodologically heterogeneous, with no studies from low-resource settings. Further research is needed in these underrepresented regions.

## 1. Introduction

Mental health challenges represent a major global public health concern. According to the World Health Organization, nearly 1 in 7 (1.1 billion) people worldwide were living with a mental disorder in 2021, with anxiety and depressive disorders being the most prevalent conditions globally (WHO, 2025). Recent Global Burden of Disease analyses confirm that mental disorders among adolescents and young adults reached an estimated 278.98 million prevalent cases globally in 2021, with anxiety and depressive disorders showing the most significant increases during the COVID-19 pandemic (Z. Wang et al., 2025). These escalating rates underscore the urgent need for scalable, accessible mental health interventions.

Exposure to natural environments has long been recognized as beneficial for mental health; however, access to restorative natural settings is often constrained by geographical, physical, or socioeconomic barriers (Y. Yang et al., 2023). Rapid urbanization and lifestyle changes have progressively reduced opportunities for everyday interaction with natural environments, potentially contributing to rising psychological distress and diminished well-being.

Parallel to these developments, the field of positive psychology has shifted the focus of mental health research from solely alleviating psychopathology to understanding and promoting human flourishing. The theoretical framework proposed by Seligman (2011) emphasizes multidimensional well-being through the PERMA model—positive emotion, engagement, relationships, meaning, and accomplishment. This perspective highlights the importance of cultivating positive psychological constructs such as resilience, compassion, gratitude, hope, self-efficacy, optimism, meaning in life, flow, and character strengths, which collectively contribute to enhanced well-being and life satisfaction (Fredrickson, 2001; Seligman, 2011). Consequently, identifying scalable interventions capable of fostering such positive psychological outcomes has become a central priority in contemporary mental health research.

The beneficial relationship between nature exposure and psychological well-being is supported by several complementary theoretical frameworks. The biophilia hypothesis proposes that humans possess an innate affinity for the natural world as a result of evolutionary adaptation (D. S. Jones & Roös, 2023; Kellert & Wilson, 1995; Li et al., 2021). In addition, Attention Restoration Theory suggests that natural environments facilitate recovery from mental fatigue by replenishing depleted attentional resources (Kaplan, 1995). Complementing this perspective, Stress Reduction Theory posits that exposure to natural settings activates parasympathetic responses that reduce physiological stress and emotional arousal (Ulrich, 1984; Ulrich et al., 1991). Together, these frameworks provide a theoretical foundation explaining how interactions with nature can promote emotional regulation, cognitive restoration, and psychological well-being (Depledge et al., 2011; Gaekwad et al., 2022).

Recent technological advancements have catalyzed the emergence of digital and virtual nature-based interventions designed to replicate aspects of natural environments through immersive or screen-based media. The number of studies examining immersive virtual nature and related technologies has grown rapidly in recent years (Brambilla et al., 2024). Virtual reality, 360° videos, smartphone applications, and digital nature imagery have emerged as accessible, scalable, and convenient modalities for delivering nature-based interventions, particularly for individuals with limited access to natural environments due to physical, geographical, or socioeconomic barriers (Finkler et al., 2025; Hubbard et al., 2025; Kumpulainen et al., 2024; McEwan et al., 2019; McEwan et al., 2020). These technologies allow users to experience simulated natural landscapes through immersive head-mounted displays, interactive environments, or mobile applications, thereby expanding the potential reach of nature-based therapeutic approaches. However, access to digital nature may considerably vary across contexts. While digital technologies offer potential for reaching underserved populations, their effectiveness depends on addressing multiple dimensions of the digital divide. Access to appropriate digital devices, reliable internet connectivity, and adequate digital literacy are foundational requirements for engaging with virtual nature interventions (Schäfer et al., 2024). Affordability of hardware and data plans, availability of technical infrastructure, and the cultural acceptability of digital mental health approaches further influence whether these interventions can be effectively implemented in diverse settings (Saboor et al., 2024). These considerations are particularly crucial in some low-resource settings, including parts of South Asia and Africa, where limited infrastructure and economic constraints may restrict the reach of digital nature interventions.

Accumulating evidence suggests that digital nature exposure is associated with meaningful psychological benefits, including reductions in anxiety, stress, and depressive symptoms, as well as improvements in mood, positive affect, restorativeness, and nature connectedness (Chen et al., 2025; Fan & Baharum, 2024; Hubbard et al., 2025; Spano et al., 2023). Beyond symptom reduction, digital nature experiences have also been linked to positive psychological capacities central to human flourishing, such as compassion, resilience, and prosocial behavior (Kang et al., 2023; Yao & Li, 2025). These findings position digital nature as a potentially promising complement to, or substitute for, physical nature exposure in contexts where access is limited.

Despite the growing body of research on digital nature interventions, several important gaps remain. Existing studies employ a wide range of technologies, intervention designs, and outcome measures, making it difficult to synthesize findings regarding their impact on positive psychological outcomes. Much of the literature has traditionally focused on reducing stress, anxiety, or negative affect, while comparatively fewer studies examine how digital nature experiences foster resilience, life satisfaction, compassion, prosociality, or nature connectedness. In addition, the psychological mechanisms underlying these effects—such as nature connectedness, perceived restorativeness, or esthetic appreciation—are often insufficiently explored. Cultural variations in well-being also remain underexamined, as collectivist societies may prioritize social harmony over individual happiness, potentially influencing how positive psychological outcomes are experienced and measured (Gu et al., 2023).

Given the rapid expansion of digital technologies and increasing interest in nature-based approaches to mental health, a comprehensive synthesis is needed to clarify the scope and potential of digital nature therapy for enhancing positive psychological outcomes. The present scoping review aims to systematically map and synthesize existing research on digital and virtual nature interventions, focusing on their effects on positive psychological constructs. Specifically, the review seeks to identify and characterize intervention types, examine the psychological outcomes addressed, explore underlying theoretical frameworks and mechanisms, and identify gaps to guide future research, policy, and practice. By providing a comprehensive overview, this review aims to advance understanding of how digitally mediated nature experiences may support human flourishing and inform the development of innovative, scalable interventions within digital mental health.

### 1.1. Operational Definitions

#### 1.1.1. Digital Nature

Digital nature refers to digitally mediated representations of natural environments and elements, such as forests, lakes, mountains, landscapes, gardens, and nature sounds (Kotera et al., 2026). These are experienced through digital platforms or devices, including virtual reality, smartphones, applications, or other digital media, either as a structured intervention or through everyday digital engagement, as long as the nature experience itself happens through a digital medium rather than in-person. Digital nature does not include people’s reflections on real, physical nature visits shared through digital channels.

#### 1.1.2. Positive Psychological Outcomes

Positive psychological outcomes refer to beneficial emotional, cognitive, and well-being-related states (Kotera et al., 2024) resulting from exposure to digital nature in this review. These outcomes encompass two conceptually distinct categories, as measured through validated tools or self-report: (a) increases in positive states such as positive affect, relaxation, well-being, mood, nature connectedness, resilience, and attentional restoration; and (b) decreases in negative states such as stress, anxiety, negative affect, and psychological distress. Throughout this review, these two categories are discussed separately to maintain conceptual clarity.

## 2. Methods

This scoping review followed a structured framework aligned with PRISMA-ScR guidelines (Tricco et al., 2018; Appendix A). Although we did not register a formal review protocol in a public database, the methodological approach was pre-specified, including eligibility criteria, search strategy, study selection, data extraction, and synthesis procedures, to ensure transparency and reproducibility.

This review was conducted as a scoping review rather than an umbrella review or evidence map; consistent with scoping review methodology, it aims to map the breadth and characteristics of the available evidence on digital nature and positive psychological outcomes, rather than to pool effect sizes across reviews or to formally assess intervention efficacy.

### 2.1. Eligibility Criteria

We included studies that examined digital or technology-mediated interventions designed to enhance psychological well-being, emotional regulation, or mental health outcomes through nature content (as operationally defined in Section 1.1) such as nature-related videos, pictures, or sounds, across diverse populations, including healthy and clinical populations. Eligible studies were published in English and included original research articles (both quantitative and qualitative studies), pilot studies, experimental studies (randomized controlled trials), mixed-method studies, and reviews (systematic reviews or meta-analyses, scoping reviews, conceptual reviews, mini-reviews), or conceptual articles, which were published in peer-reviewed journals. We included only studies for which the full text was available.

### 2.2. Exclusion Criteria

We excluded studies published before 2020. This cutoff was chosen for two reasons. First, COVID-19 lockdowns restricted access to outdoor nature while accelerating adoption of digital health technologies, prompting researchers to test virtual nature as a substitute when real nature was inaccessible (Phillips et al., 2021; Labib et al., 2020). Second, digital nature is a recent and still-emerging construct. A rapid review published in 2021 identified only three studies that specifically examined digital nature as an intervention to improve well-being, indicating a limited evidence base in this area prior to 2020 (Van Houwelingen-Snippe et al., 2021). A pre-2020 cutoff would therefore add minimal relevant literature. We also excluded studies that did not use digital or technology-mediated interventions related to nature exposure, psychological well-being, or mental health outcomes. We excluded studies that focused solely on traditional, in-person nature interventions. We excluded studies that did not report relevant psychological, emotional, cognitive, or behavioral outcomes. We excluded non-peer-reviewed sources, conference abstracts without full texts, editorials, commentaries, preprints, books, encyclopedias and studies not written in English. A list of excluded articles, along with the corresponding reasons for exclusion, is provided on the Supplementary Material (File S1).

### 2.3. Information Sources and Search Strategy

We searched PubMed, Google Scholar, and ScienceDirect using predefined keywords and Boolean operators. The search was conducted systematically across these three databases to ensure reproducibility. Our initial search used a 2019–2025 window. However, we re-ran the search between 5 and 10 June 2026, applying a revised, fixed window of 1 January 2020 to 25 December 2025 to ensure consistency with our updated rationale.

**Figure 1:**
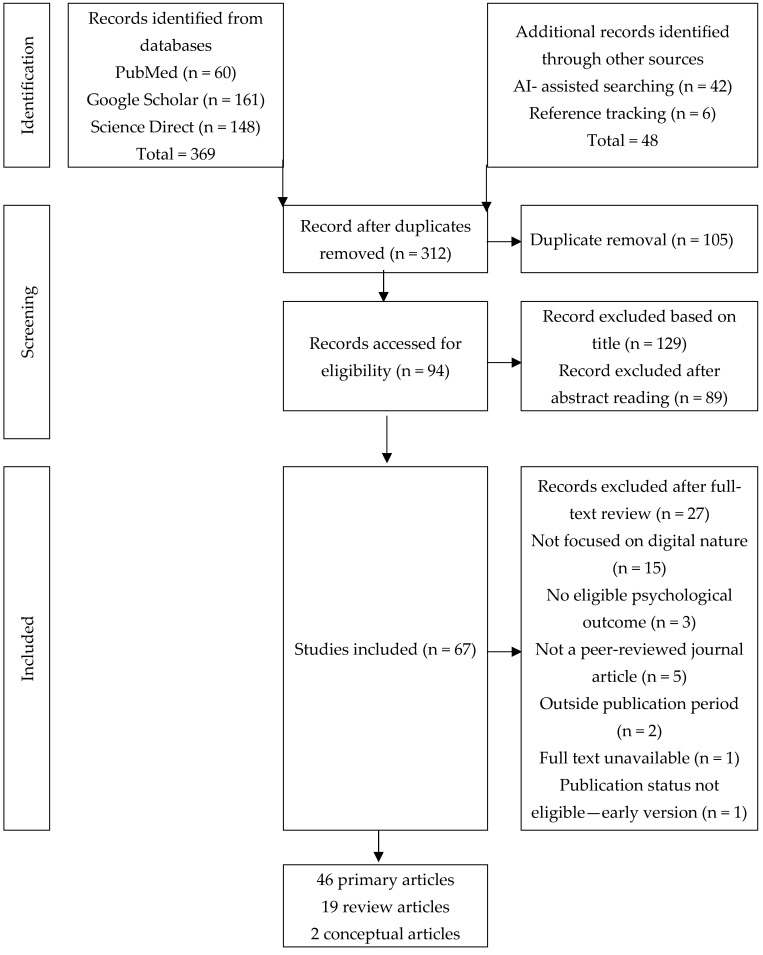
PRISMA-ScR flow diagram showing the identification, screening, eligibility, and inclusion stages of studies for the scoping review on digital nature interventions and positive psychological outcomes. Note: The details of the articles excluded after full-text review are found in the Supplementary Material (File S1).

Database-specific Boolean strings were constructed by combining exposure-related terms (e.g., “digital nature,” “virtual nature,” “immersive nature,” “VR nature”) with terms for positive psychological outcomes (e.g., resilience, optimism, compassion, well-being, connectedness, meaning in life), adapted to each database’s supported syntax and field tags (H. Yang & Lee, 2018). Appropriate filters (publication date, language, full-text availability, article type, and exclusion of preprints) were applied in each database to control search noise. These filters were applied to ensure the inclusion of peer-reviewed, accessible, and methodologically relevant studies while maintaining feasibility. Database-specific record counts, including the number of results returned by each search strategy, are presented in the PRISMA-ScR flow diagram (Figure 1). Complete Boolean strings, filters, and access dates for each database are provided in the Supplementary Material (File S1). An AI-assisted tool (Claude Sonnet 4.6, Anthropic) was used as a supplementary aid to identify potentially relevant articles that might not have been found through database searches. Claude was prompted using predefined search terms and three rounds of search keyword refinement to suggest potentially relevant articles. All AI-suggested records were manually verified by accessing the original articles through the provided links. Only records that could be verified through direct access to the published source were considered. Potentially hallucinated or non-verifiable outputs were excluded. The specific prompts used are provided in the Supplementary Material (File S1). All AI-generated references and outputs were manually checked for accuracy by the authors.

### 2.4. Selection of Sources of Evidence

All identified studies were screened independently by two reviewers based on titles and abstracts. Full texts were then assessed for eligibility according to the predefined criteria. Disagreements were resolved through discussion, with a third reviewer consulted when consensus could not be reached. This process ensured systematic inclusion of studies reporting on digital interventions and psychological outcomes.

### 2.5. Data Items and Data Charting Process 

We developed a standardized data extraction form to capture relevant information from each study. Two reviewers independently charted the data, including author(s), year of publication, country, study objectives, source type/design, intervention type, sample size, instruments and measures, framework, quality assessment, and major findings related to digital nature and psychological well-being. Data items were extracted according to article type, as extraction for review articles differed slightly from that for primary articles. The extracted data were cross-checked to ensure accuracy, and discrepancies were resolved by discussion among the review team. Mendeley citation manager managed the citations and referencing. No dedicated software was used for screening.

### 2.6. Critical Appraisal of Individual Sources of Evidence

A formal risk of bias assessment was not conducted, consistently with scoping review methodology. Instead, we extracted and reported the study design, sample characteristics, and methodological details to provide context regarding the robustness and generalizability of the evidence.

### 2.7. Synthesis of Results

Given the diversity of study designs, populations, and intervention types, findings were synthesized using a descriptive narrative approach consistent with the Synthesis Without Meta-analysis (SWiM) guideline (Campbell et al., 2020). Primary studies, reviews and meta-analyses, and conceptual articles were synthesized separately, to preserve the distinct evidentiary status of each category and to avoid treating empirical findings, aggregated review-level evidence, and theoretical contributions as equivalent. Within each category, studies were grouped thematically according to the psychological outcomes they addressed, such as stress reduction, anxiety and affect, restorativeness, nature connectedness, and feasibility or engagement, rather than by intervention modality alone, since multiple modalities (e.g., immersive VR, 360-degree video, smartphone applications) frequently targeted the same outcomes. This approach allowed patterns, inconsistencies, and gaps to be identified both within and across categories of evidence, while keeping empirical findings distinct from theoretical and conceptual contributions throughout the results and discussion.

Review articles were used primarily to contextualize the evidence and to provide aggregated estimates of effect where available, rather than to strengthen conclusions drawn from individual primary studies. When review-level evidence and primary study findings converged, this was noted as reinforcing the overall pattern; when they diverged, this was reported as an inconsistency requiring further investigation. The potential overlap between primary studies included in our review and those included in the reviewed meta-analyses was not formally quantified, and this is acknowledged as a limitation.

## 3. Results

### 3.1. Overview of Included Studies

#### 3.1.1. Primary Articles

We included 46 primary studies. Most used experimental designs (*n* = 17) or randomized controlled trials (*n* = 14). The rest used pre–post designs (*n* = 7), cross-sectional surveys (*n* = 3), mixed-method designs (*n* = 2), qualitative designs (*n* = 2), or a non-randomized interventional clinical trial (*n* = 1). Most studies came from Europe (*n* = 25), with fewer from Asia (*n* = 10), North America (*n* = 6), Oceania (*n* = 3), and Africa (*n* = 1). One study sampled participants across multiple continents (Supplementary File S1).

Sample sizes varied considerably, ranging from very small qualitative/feasibility samples (*n* = 7) to large-scale survey and analytic datasets (*N* = 8752) (Lundstedt et al., 2021; Smalley et al., 2023). Populations included healthy adults and university students (Mostajeran et al., 2023a; J. Kim et al., 2025); adolescents (Owens & Bunce, 2023; Fahey, 2025); older adults and residential aged-care populations (Appel et al., 2020; Brimelow et al., 2020; Lundstedt et al., 2021; Clemente et al., 2024); and clinical populations including psychiatric outpatients, individuals with depression, cancer patients, dementia patients, and patients with gambling disorder or generalized anxiety disorder (Veling et al., 2021; B. Kim et al., 2025; Zeng et al., 2025; Matsangidou et al., 2025; Ochiai et al., 2023; T. Wang et al., 2020).

Digital nature interventions spanned a wide technological range, including immersive head-mounted display (HMD)-based virtual reality (360-degree video and computer-generated/CG-VR), 2D screen-based nature videos and images, multisensory projections incorporating scent and sound, smartphone applications, digital therapeutic gardens, and auditory-only nature soundscapes, which are included as they meet the operational definition of digitally mediated nature experience (van Houwelingen-Snippe et al., 2020b; Yao & Li, 2025; Lee et al., 2025; McEwan et al., 2020; Ojala et al., 2022). The most frequently assessed outcomes were stress reduction, anxiety and negative affect reduction, positive affect and mood enhancement, restorativeness, and nature connectedness. Less frequently measured outcomes included attention restoration, self-compassion, vitality, and resilience-related constructs. These outcomes are grouped into two conceptually distinct categories: (a) reductions in negative states, such as stress, anxiety, depressive symptoms, and pain; and (b) increases in positive psychological constructs, such as positive affect, restorativeness, resilience, and nature connectedness. Both categories are reported throughout this review.

#### 3.1.2. Review Articles

We included nineteen review articles. These comprised systematic reviews and meta-analyses (n = 7), systematic reviews without meta-analysis (n = 6), a systematic qualitative review, a systematic literature review, scoping reviews (n = 2), a mini-review, and a narrative review (Figure 1; Supplementary File S1). These reviews collectively synthesized evidence on virtual and immersive nature exposure and its relationship to stress, anxiety, depression, mood, pain, nature connectedness, and well-being. Several reviews specifically examined nature-based VR interventions in clinical populations, including VR-assisted mindfulness training incorporating natural environments and VR-based therapy for patients with paranoia and dementia. Review articles were used primarily to contextualize the broader evidence base and to provide aggregated effect estimates, not as independent evidence sources for individual outcome claims.

#### 3.1.3. Conceptual Articles

We included two conceptual/theoretical articles. Williams and Langley (2021) offered a theoretical perspective on immersive virtual nature art and its potential to support well-being through biophilic, restorative design principles. Fuentes et al. (2024) proposed a conceptual model of digital placemaking in nature, reframing human–nature relationships in urban contexts through digital extensions of physical space in service of well-being.

### 3.2. Thematic Analysis of Primary Articles

#### 3.2.1. Theme 1: Stress Reduction and Physiological Relaxation

Digital nature was associated with reductions in perceived and physiological stress across diverse delivery formats. Office-based virtual nature breaks were associated with lowered stress and reduced heart rate (Ojala et al., 2022; T. Wang et al., 2020). VR relaxation sessions were linked with reduced stress in psychiatric outpatients and students (Veling et al., 2021; Ahn et al., 2025; J. Kim et al., 2025). Nature soundscapes were associated with reduced stress markers in gambling-disorder patients and in a crossover study of healthy adults (Ochiai et al., 2023; Kumpulainen et al., 2025). Immersive computer-generated nature was associated with lowered stress and improved cognition (Mostajeran et al., 2023a). Some studies found physiological support for these associations, including reduced prefrontal brain activity linked to stress (Ochiai et al., 2023) and higher alpha-wave activity linked with relaxation (T. Wang et al., 2020; Chan et al., 2021). Benefits sometimes extended beyond the session; VR relaxation was connected with improved sleep quality at four-week follow-up (Ahn et al., 2025).

Not all findings were positive. A VR-based emotion-focused therapy did not show robust, statistically significant reductions in stress or self-compassion (Halamová et al., 2025). In one study, unnatural motion effects, not naturalistic visual scenes, produced the strongest physiological recovery (Fang et al., 2025), suggesting that stress relief does not always depend on how realistic the digital environment appears.

#### 3.2.2. Theme 2: Reduction in Anxiety, Depression

Several studies examined reductions in anxiety and depressive symptoms. Daily virtual nature exposure was linked with reduced worry, panic, and rumination in college students (Browning et al., 2023). VR meditation was connected with reduced state anxiety and perceived stress in university students (J. Kim et al., 2025). VR-based mindfulness–cognitive therapy incorporating natural virtual environments showed preliminary promise for individuals with depression (B. Kim et al., 2025). Online forest bathing in VR was associated with reduced anxiety, rumination, and Long COVID symptoms (McEwan et al., 2022). In clinical populations, VR was associated with reduced anxiety, aggression, and depression-related behavioral symptoms in dementia patients (Matsangidou et al., 2025), and with reduced anxiety symptoms in hospice caregivers (Lehto et al., 2025). A digital therapeutic garden was associated with lower stress in individuals with higher baseline depression or anxiety (Lee et al., 2025).

#### 3.2.3. Theme 3: Positive Affect, Mood, and Well-Being

Many studies reported improved well-being following digital nature exposure. Digital nature through VR exposure, including 360-degree VR nature, was associated with improved mood, positive affect, attention and sleep quality in adolescents and students (Owens & Bunce, 2023; Finkler et al., 2025; Reese et al., 2022; Appel et al., 2020; Ahn et al., 2025). Underwater VR was associated with reduced boredom, while interactive CG-VR was associated with greater improvements in positive affect than passive 2D video (Yeo et al., 2020). Both outdoor and VR nature exposure were associated with increased positive affect compared to indoor controls (Browning et al., 2020; Reese et al., 2022). Digital forest was associated with higher self-compassion (Szitás et al., 2025). Exposure to virtual nature images was linked with improved positive affect and well-being, particularly when paired with a sense of self-agency (Donald & Egboluche, 2023). A smartphone nature application was connected with improved well-being and nature connectedness (McEwan et al., 2020).

Findings were less consistent in aged-care settings. VR nature was linked with reduced sadness and worry in older adults with cognitive or physical impairments (Appel et al., 2020; Lundstedt et al., 2021). In residential aged care, VR was associated with reduced apathy but showed no significant effect on mood (Brimelow et al., 2020), illustrating that benefits do not uniformly extend across all outcomes or settings.

#### 3.2.4. Theme 4: Restorativeness, Attention, and Cognitive Recovery

A subset of studies focused on attention restoration and cognitive functioning. Virtual natural environments were connected with greater restorativeness and subjective vitality compared to virtual urban settings (Theodorou et al., 2023; Boffi et al., 2022). Adding virtual plants to a VR environment was linked with improved positive affect, attentive coping, and sense of presence (Mostajeran et al., 2023b). Immersive CG nature was associated with improved executive functioning and memory alongside enhanced restorativeness (Mostajeran et al., 2023a). Forest and water videos accompanied by sound were connected with stronger restorative recovery than sound-only or silent conditions (Ojala et al., 2022). Natural sounds were rated as more restorative than music, and positive personal memories were associated with greater perceived restoration (Smalley et al., 2023).

#### 3.2.5. Theme 5: Nature Connectedness and Immersion Effects

Nature connectedness appeared as both an outcome and a mediating pathway. Digital nature videos were associated with increased community connectedness, with greater distance from physical nature predicting higher loneliness (van Houwelingen-Snippe et al., 2020a). Virtually embodying nature (growing as a tree) was associated with increased connectedness, with compassion mediating this relationship (Spangenberger et al., 2025). Interactive CG-VR was connected with greater connectedness than passive 2D video (Yeo et al., 2020). Connectedness mediated the relationship between exposure to a virtual Arctic environment and vigor in older adults (Clemente et al., 2024). Walking in virtual nature was connected with increased nature connection in both young adults and seniors (Chan et al., 2021; Finkler et al., 2025).

Awe-related experiences also emerged as relevant. Awe-inspiring virtual nature was associated with increased socially oriented pro-environmental behavior, though not with personally oriented behavior (Chirico et al., 2023). Awe-inspiring virtual environments were associated with greater fascination and joy in aged-care residents (Lundstedt et al., 2021).

#### 3.2.6. Theme 6: Modality, Fidelity, and Moderating Variables

Delivery modality shaped the pattern of outcomes. Interactive CG-VR was associated with greater improvements in positive affect and nature connectedness compared to 2D television (Yeo et al., 2020). Immersive VR was linked with a stronger sense of presence than 360-degree video during exercise, though 360-degree video was rated as more pleasant and enjoyable (L. Jones & Wheat, 2023). Similarly, 3D VR models produced stronger presence than 360-degree video during green exercise (Litleskare et al., 2022). These findings suggest that presence and enjoyment are distinct outcomes, and that the optimal modality depends on the specific goal of the intervention.

Population characteristics also moderated outcomes. Male participants were more receptive to digital forest bathing, while female participants showed skepticism despite stronger predispositions toward traditional forest immersion (Oe et al., 2025). Short exposures, sometimes as brief as six minutes, were sufficient to produce measurable benefits (Owens & Bunce, 2023; Browning et al., 2020; Appel et al., 2020), while benefits grew stronger with repeated or daily exposure in other contexts (Ahn et al., 2025; Browning et al., 2023; Appel et al., 2022). Scene characteristics also mattered: tended (cultivated) scenes were associated with greater social aspiration than wild scenes, while dense scenes were rated as more fascinating than spacious ones under social viewing conditions (van Houwelingen-Snippe et al., 2020b; van Houwelingen-Snippe et al., 2020a).

#### 3.2.7. Theme 7: Feasibility and Engagement

A smaller group of studies focused on feasibility and acceptability rather than direct psychological outcomes. VR therapy for veterans with dementia was found feasible to deliver during behavior-triggering moments, with all participants choosing to continue with additional sessions (Appel et al., 2022). A VR stress-management platform for a defense workforce was feasible across multiple sites and associated with high user engagement (Kluge et al., 2023). Additional studies also reported high levels of immersion, presence, enjoyment, or participant engagement during digital nature interventions, supporting the acceptability of these approaches (Brimelow et al., 2020; L. Jones & Wheat, 2023; Lehto et al., 2025; Litleskare et al., 2022). These findings suggest that engagement and acceptability are meaningful outcomes in their own right, not merely preconditions for measuring psychological effects.

A wide range of primary studies using diverse methodologies has established a clear, beneficial association between digital nature interventions and positive psychological and physiological outcomes (Table 1).

### 3.3. Thematic Analysis of Review Articles

Review articles were used to contextualize patterns identified in the primary literature and to provide aggregated effect estimates where available. The potential overlap between primary studies included in this review and those synthesized within the included reviews was not formally quantified and is acknowledged as a limitation (see Section 4.6).

#### 3.3.1. Theme 1: Evidence for Stress, Anxiety, and Depression Reduction

Several reviews reported that digital nature was associated with reductions in stress, anxiety, and depression. One meta-analysis reported a large effect size for anxiety reduction and moderate effects for stress and depression across 24 studies, recommending brief 10–15 min sessions for anxiety relief (Chen et al., 2025). Another review found beneficial pre–post effects for mood, anxiety, stress, and cognition in higher education students, though no significant advantage of VR nature over flat-screen nature for anxiety was found (Hubbard et al., 2025). Two reviews concluded that virtual nature produces psychological and psychophysiological benefits broadly comparable to real nature (Spano et al., 2023; Abdullah et al., 2021). Digital nature matched real nature for stress recovery when content was held constant (Fan & Baharum, 2024). One review extended these findings to patient populations, reporting that VR nature was associated with improved pain, anxiety, and fear alongside reduced heart rate and blood pressure (Wen et al., 2024).

Not all reviews found consistent results. One review found that virtual nature reliably reduced negative affect but reported inconsistent results for positive affect and physiological stress responses (Frost et al., 2022). A rapid review on older adults concluded that evidence for digital nature remains limited and called for further research (Van Houwelingen-Snippe et al., 2021). A scoping review on 360-degree VR for older adults found the approach feasible and well-received, but noted that small samples and design heterogeneity limited generalizability (Restout et al., 2023).

#### 3.3.2. Theme 2: Multisensory Immersion and Modality Comparisons

Some reviews examined how sensory richness and delivery modality shaped outcomes. The addition of olfactory stimuli to multisensory digital nature setups produced effects comparable to those of conventional natural environments (Lopes & Falk, 2024). Interactive CG-VR was suggested to offer a stronger sense of presence than 360-degree video (Li et al., 2021). Immersive virtual nature was associated with greater increases in nature connectedness than non-immersive formats, particularly when access to real nature was constrained (Brambilla et al., 2024).

#### 3.3.3. Theme 3: Mechanisms and Theoretical Synthesis

One scoping review offered a theoretical framework for why VR might support well-being. Synthesizing 187 articles, it proposed that Place Illusion, the sense of physically being present in a virtual scene, primarily enhances subjective well-being through restoration and awe, while Plausibility and Body Ownership illusions link more strongly to general psychological well-being (Lin et al., 2025). This framework provides a possible explanation for why presence-focused and embodiment-focused interventions do not always produce the same type or magnitude of benefit.

#### 3.3.4. Theme 4: Effectiveness in Clinical and Vulnerable Populations

Several reviews focused on clinical applications of VR. A review of 721 articles reported strong support for VR-based exposure therapy for anxiety and paranoia, and found that VR-based assessment could distinguish dementia from healthy cognitive functioning (Wiebe et al., 2022). Another review found VR effective for improving social participation and reducing anxiety and paranoid symptoms in patients with paranoia (Monaghesh et al., 2022). VR-assisted mindfulness training was reported to outperform conventional mindfulness training for state mindfulness, anxiety, depression, and sleep quality (Ma et al., 2023). VR programs combining nature, mindfulness, and compassion were described as an accessible option for older adults, though the authors called for more rigorous empirical validation (Sadowski & Khoury, 2022). VR was associated with anxiety reduction in older adults, though effects on pain management were mixed and occasionally non-significant (Corbel et al., 2025).

#### 3.3.5. Theme 5: Broader Digital Well-Being Interventions

One review examined digital positive psychology interventions more broadly. It found that web-based and smartphone-delivered programs were associated with significant improvements in purpose, gratitude, and hope, alongside reductions in stress in children, adolescents, and young adults (Saboor et al., 2024). This situates digital nature within a wider trend toward technology-delivered tools for psychological well-being.

### 3.4. Thematic Analysis of Conceptual Articles

Two conceptual articles were included to provide theoretical frameworks for interpreting the empirical findings. Williams and Langley (2021) explored perspectives on immersive virtual nature art and suggested that it shows potential to support well-being through restorative immersive experiences based on biophilic design. Similarly, Fuentes et al. (2024) conceptualized digital placemaking in nature and proposed a model to reframe human–nature relationships in cities through digital extensions of physical space for well-being. Importantly, these articles propose theoretical mechanisms and models; they do not demonstrate empirical effects but rather offer frameworks for understanding the pathways through which digital nature may influence psychological outcomes.

#### Summary

Across the primary studies, digital nature was associated with reductions in stress, anxiety, and depressive symptoms, and with improvements in positive affect, mood, restorativeness, and nature connectedness. These patterns were observed across healthy adults, older adults, and clinical populations. However, not every finding was positive: some studies found no significant effect on stress or mood (Halamová et al., 2025; Brimelow et al., 2020), showing that benefits vary across outcomes, populations, and delivery formats. The type of technology influenced results: interactive VR was generally associated with greater gains in affect and connectedness than passive video, though higher presence did not always correspond with greater enjoyment or benefit. A small number of studies focused on feasibility and engagement, finding digital nature interventions well-accepted even in challenging settings such as dementia care and defense workforces.

At the review level, meta-analyses reported large effects for anxiety reduction and moderate effects for stress and depression, often achievable within 10–15 min sessions. However, some reviews found mixed results for positive affect and physiological outcomes, and one rapid review described the evidence base as still limited for older adult populations. One review proposed that the sense of presence (Place Illusion) drives restorative and awe-related well-being, while embodiment-related illusions are more closely linked to broader psychological well-being (Lin et al., 2025).

The two conceptual articles provided theoretical grounding through biophilic design and digital placemaking frameworks, offering complementary explanations for why digitally mediated nature can support well-being even without physical access to natural environments.

## 4. Discussion

### 4.1. Overview of Findings

This scoping review synthesized evidence from 46 primary studies, 19 review articles, and two conceptual papers to examine the effects of digital nature interventions on positive psychological outcomes. The evidence was synthesized separately according to study type to maintain methodological clarity and minimize overlap between primary studies and review articles. Overall, the findings indicate that digital nature interventions are consistently associated with beneficial psychological outcomes, particularly through reductions in stress, anxiety, and negative affect, together with improvements in positive affect, well-being, restorativeness, nature connectedness, and other positive psychological resources. However, substantial heterogeneity in intervention modalities, participant characteristics, outcome measures, and study designs limits direct comparisons across studies and precludes firm conclusions regarding the optimal intervention characteristics.

Although the evidence was synthesized separately according to study type, the three bodies of literature converged on a broadly consistent pattern. Primary studies provided direct empirical evidence of psychological benefits, review articles largely reinforced these findings while highlighting methodological limitations, and conceptual papers offered theoretical explanations for the observed effects. Together, these complementary sources strengthen confidence in the potential of digital nature interventions while emphasizing the need for more rigorous and standardized research. However, because some primary studies may also have been included in the review articles, this convergence should be interpreted cautiously, as part of the observed consistency may reflect overlap in the underlying evidence rather than entirely independent sources of support.

### 4.2. Interpreting the Primary Article Themes

The primary studies consistently demonstrated that digital nature interventions—including immersive virtual reality, smartphone applications, online forest bathing, digital therapeutic gardens, and nature soundscapes—were associated with beneficial psychological outcomes across both healthy and clinical populations. The most consistent findings involved reductions in stress, anxiety, and negative affect alongside improvements in positive affect, relaxation, and psychological well-being (Ahn et al., 2025; Browning et al., 2023; J. Kim et al., 2025; McEwan et al., 2022; Veling et al., 2021). These findings are consistent with the Stress Recovery Theory and Attention Restoration Theory, which propose that exposure to natural environments facilitates emotional recovery and restoration of cognitive resources.

Importantly, the benefits of digital nature extended beyond reducing psychological distress. Several studies reported improvements in life satisfaction, vitality, self-compassion, nature connectedness, social connectedness, executive functioning, and memory (Lee et al., 2025; Mostajeran et al., 2023a; Owens & Bunce, 2023; Spangenberger et al., 2025; Szitás et al., 2025), suggesting that digital nature may foster psychological flourishing in addition to alleviating distress. Nature connectedness frequently emerged as both an outcome and a potential mechanism underlying these benefits. Connectedness mediated the relationship between digital nature exposure and vigor (Clemente et al., 2024), whereas compassion mediated the relationship between virtual nature embodiment and nature connectedness (Spangenberger et al., 2025). Furthermore, awe-inspiring digital nature promoted socially engaging pro-environmental behavior, with positive affect and pro-environmental attitudes positively associated with this response (Chirico et al., 2023), indicating that digital nature may also promote broader psychosocial engagement.

The findings further suggest that intervention effectiveness depends not only on technological sophistication but also on intervention content and user experience. Interactive and immersive environments generally enhanced presence and engagement (L. Jones & Wheat, 2023; Litleskare et al., 2022), while interactive computer-generated virtual nature also increased nature connectedness (Yeo et al., 2020). However, greater immersion did not consistently translate into superior psychological outcomes. For example, 360° videos were perceived as more pleasant and enjoyable than fully immersive virtual reality in some contexts (L. Jones & Wheat, 2023), whereas physiological recovery was influenced by virtual motion characteristics rather than environmental realism alone (Fang et al., 2025). These findings suggest that multiple design characteristics may contribute to intervention effectiveness beyond immersion itself.

Although most studies reported favorable outcomes, inconsistencies were also evident. Some interventions demonstrated limited or non-significant effects on mood, self-compassion, or longer-term anxiety outcomes (Brimelow et al., 2020; Halamová et al., 2025; Veling et al., 2021). Additionally, although digital nature significantly improved mood and prefrontal cortical activity, not all physiological indicators changed consistently, with no significant effect observed for heart rate variability in one study (Ochiai et al., 2023). Studies employing repeated or daily digital nature exposure also reported sustained or cumulative psychological benefits (Ahn et al., 2025; Browning et al., 2020), although none directly compared repeated and single-session interventions. Consequently, the optimal intervention dosage and duration remain uncertain.

### 4.3. Interpretation of Review-Level Evidence

The review articles generally reflected patterns similar to those observed in the primary studies, indicating that digital nature interventions are associated with reductions in anxiety, stress, depression, and negative affect while promoting relaxation, restoration, and psychological well-being (Abdullah et al., 2021; Chen et al., 2025; Hubbard et al., 2025; Spano et al., 2023). Meta-analytic evidence further suggests that relatively brief interventions may effectively reduce anxiety and that digital nature can produce stress recovery comparable to physical nature when intervention content is similar (Chen et al., 2025; Fan & Baharum, 2024). Reviews also identified potential benefits for pain management, mindfulness, cognition, and social participation across several clinical populations (Corbel et al., 2025; Ma et al., 2023; Monaghesh et al., 2022; Wiebe et al., 2022).

Despite these encouraging findings, the review literature consistently highlighted methodological limitations. Inconsistent evidence was reported for positive affect, physiological stress responses, and differences between immersive and non-immersive digital nature interventions (Frost et al., 2022; Hubbard et al., 2025). Furthermore, considerable heterogeneity in intervention protocols, technologies, participant populations, and outcome measures limits confidence in pooled estimates and highlights the need for more rigorous, standardized, and adequately powered studies (Sadowski & Khoury, 2022; Van Houwelingen-Snippe et al., 2021).

### 4.4. Interpretation of Conceptual Contributions

The conceptual papers complement the empirical evidence by providing theoretical perspectives on the role of digital nature in promoting psychological well-being. Fuentes et al. (2024) proposed that digital extensions of urban environments may strengthen human–nature relationships when direct contact with nature is limited. Similarly, Williams and Langley (2021) suggested that immersive virtual nature designed according to biophilic principles may facilitate restorative experiences and emotional well-being. Although these frameworks require further empirical validation, they are broadly consistent with the observed improvements in restoration, nature connectedness, and well-being identified across the empirical literature.

### 4.5. Limitations of the Evidence Base

Several limitations of the available evidence and the present review should be acknowledged. Many primary studies had small samples, often under 100 participants, and some used single-group designs without a control condition. This limits how confidently we can draw cause-and-effect conclusions. The search was limited to PubMed, Google Scholar, and ScienceDirect, which may have excluded relevant studies from other databases. The use of filters (full-text availability, open access, English language, and exclusion of preprints) may have introduced selection and availability bias (Kotera et al., 2022). Follow-up periods were usually short; only a few studies checked whether benefits lasted beyond the session itself (Ahn et al., 2025). Therefore, the long-term sustainability of these psychological benefits remains uncertain.

Outcome measures, intervention content, technological platforms, and delivery methods varied widely across studies. Additionally, the included studies employed diverse psychological outcome measures, which may have limited direct comparison across studies despite assessing conceptually related outcomes. Although this heterogeneity reflects the breadth of digital nature interventions, it limits direct comparison across studies and the identification of optimal intervention characteristics, a point also raised by several of the included reviews (Frost et al., 2022). Because primary studies and reviews were charted and reported separately, some primary studies may also be represented within the samples of the included reviews; this overlap was not formally identified or adjusted for, and review articles were used primarily for contextualization rather than as independent evidence sources.

Because no formal risk-of-bias or quality assessment was conducted, consistently with standard scoping review methodology, the relative methodological strength of the included studies could not be weighted in this synthesis. Formal inter-rater agreement statistics (e.g., Cohen’s kappa [κ]) were not calculated for the screening or data-charting process; disagreements were instead resolved through discussion, which may limit the assessment of screening reliability. This review included studies published between 2020 and 2025, which may have excluded earlier evidence relevant to the development of this field. Finally, most evidence came from Europe and parts of Asia, so whether these findings generalize to other regions and cultural contexts remains unknown.

### 4.6. Implications and Future Directions

The evidence for stress reduction, mood improvement, and connectedness suggests that digital nature tools could help in places where real nature is hard to reach. This includes hospitals, aged-care homes, offices, and remote settings, a possibility directly supported by findings involving hospice caregivers (Lehto et al., 2025), aged-care residents (Brimelow et al., 2020; Lundstedt et al., 2021), and psychiatric patients (Veling et al., 2021). At the same time, the inconsistencies found across this review suggest that future studies should ask more specific questions. Instead of asking whether digital nature works in general, researchers might ask which features, such as interactivity, scent, embodiment, or plant density, produce which specific benefits, for which specific groups of people. This indicates a need for more cross-cultural studies in this area evaluating their experiences (Kotera et al., 2025).

Better methods would also help. These include more consistent outcome measures, longer follow-up periods, and research drawn from a wider range of countries and cultures. Feasibility and engagement also deserve more direct attention in future research, not just as a step before measuring other outcomes, but as goals in their own right. Finally, the two conceptual frameworks in this review, biophilic design and digital placemaking, have not yet been tested directly against outcome data. This is a clear opportunity for future studies to connect theory and evidence more closely.

## 5. Conclusions

This scoping review synthesized evidence from 46 primary studies, 19 reviews and meta-analyses, and two conceptual articles to map the effects of digital nature interventions on positive psychological outcomes. Overall, the available evidence suggests a broadly positive pattern that digitally mediated nature exposure, whether delivered through immersive virtual reality, 360-degree video, smartphone applications, digital therapeutic gardens, or nature soundscapes—is associated with reductions in stress and anxiety, improvements in positive affect and mood, enhanced perceived restorativeness, and greater nature connectedness across healthy, older adult, and clinical populations. The two conceptual contributions further suggest that the psychological benefits may be understood through complementary theoretical perspectives, including biophilic design and digital placemaking, which provide plausible explanations for how digitally mediated nature experiences may support well-being when access to physical natural environments is limited.

Although the overall pattern of findings is encouraging, benefits were not uniform across all outcomes or populations, and intervention modality and immersion level influenced specific effects. Furthermore, the evidence should be interpreted cautiously because much of it is geographically concentrated in Europe and parts of Asia, methodologically diverse, and potentially affected by overlap between primary studies and the included review articles. Accordingly, consistency between primary studies and review articles should be viewed as complementary rather than wholly independent evidence. Future research should prioritize standardized outcome measures, adequately powered comparative study designs, longer-term follow-up, broader geographic representation, and greater outcome-specific precision to strengthen the evidence base and better establish the effectiveness of digital nature interventions.

## Figures and Tables

**Table 1:** Summary of thematic evidence from primary studies.

Thematic Domain	Primary Outcome	Direction	Key Primary Studies
Stress Reduction	Reduction in perceived and physiological stress	Beneficial association	(Ojala et al., 2022; Veling et al., 2021; Ahn et al., 2025; Mostajeran et al., 2023a; Chan et al., 2021)
Anxiety and Depression	Reduction in anxiety, worry, rumination, and depressive symptoms	Beneficial association	(Browning et al., 2023; J. Kim et al., 2025; McEwan et al., 2022; Matsangidou et al., 2025)
Positive Affect and Mood	Improvement in positive affect, mood, and well-being	Beneficial association	(Yeo et al., 2020; Owens & Bunce, 2023; Browning et al., 2020; Donald & Egboluche, 2023)
Restorativeness and Cognitive Recovery	Enhancement of perceived restorativeness, attention, and executive function	Beneficial association	(Theodorou et al., 2023; Mostajeran et al., 2023b; Mostajeran et al., 2023a)
Nature Connectedness	Increase in felt connection to nature	Beneficial association	(Yeo et al., 2020; Chan et al., 2021; Spangenberger et al., 2025; Clemente et al., 2024; McEwan et al., 2022)
Compassion and Prosociality	Increase in compassion and pro-environmental behavior	Beneficial association	(Spangenberger et al., 2025; Chirico et al., 2023)
Social Connectedness	Increase in community connectedness and reduced loneliness	Beneficial association	(van Houwelingen-Snippe et al., 2020a; van Houwelingen-Snippe et al., 2020b; Lee et al., 2025)
Physiological Relaxation	Reduction in physiological stress markers (heart rate, brain activity, skin conductance)	Beneficial association	(Chan et al., 2021; Ochiai et al., 2023; T. Wang et al., 2020)
Feasibility and Engagement	High acceptability and user engagement	Beneficial association	(Appel et al., 2022; Kluge et al., 2023)

Note: “Beneficial association” means digital nature was associated with improvement in positive states or reduction in negative states, as appropriate to each domain. This table summarizes primary study findings only.

## Data Availability

Data will be provided by the corresponding author upon reasonable request.

## Supplementary Materials

The following supporting information can be downloaded at https://www.mdpi.com/article/10.3390/bs16071257/s1. Table S1: Systematic Search Strategy, Boolean Operators, and Article Identification Across Databases; Table S2: Excluded Full-Text Articles with Rationale for Exclusion Following Eligibility Assessment; Table S3: Characteristics of Included Primary Studies; Table S4: Characteristics of Included Review Articles; Table S5: Characteristics of Included Conceptual and Theoretical Papers.

## Abbreviations

2D	2-Dimensional
3D	3-Dimensional
CG-VR	Interactive Computer-Generated Virtual Reality
HMD	Head-Mounted Display
PRISMA	Preferred Reporting Items for Systematic Reviews and Meta-Analyses
PRISMA-ScR	Preferred Reporting Items for Systematic Reviews and Meta-Analyses Extension for Scoping Reviews
RCT	Randomized Controlled Trial
SWiM	Synthesis Without Meta-Analysis
VR	Virtual Reality

## Appendix A

**Table A1:** Preferred Reporting Items for Systematic reviews and Meta-Analyses extension for Scoping Reviews (PRISMA-ScR) Checklist.

Section	Item	PRISMA-ScR Checklist Item	Reported on Page #
**Title**			
Title	1	Identify the report as a scoping review.	Page 1
**Abstract**			
Structured summary	2	Provide a structured summary that includes (as applicable): background, objectives, eligibility criteria, sources of evidence, charting methods, results, and conclusions that relate to the review questions and objectives.	Page 1
**Introduction**			
Rationale	3	Describe the rationale for the review in the context of what is already known. Explain why the review questions/objectives lend themselves to a scoping review approach.	Page 3
Objectives	4	Provide an explicit statement of the questions and objectives being addressed with reference to their key elements (e.g., population or participants, concepts, and context) or other relevant key elements used to conceptualize the review questions and/or objectives.	Page 3
**Methods**			
Protocol and registration	5	Indicate whether a review protocol exists; state if and where it can be accessed (e.g., a Web address); and if available, provide registration information, including the registration number.	Page 4
Eligibility criteria	6	Specify characteristics of the sources of evidence used as eligibility criteria (e.g., years considered, language, and publication status), and provide a rationale.	Page 4
Information sources *	7	Describe all information sources in the search (e.g., databases with dates of coverage and contact with authors to identify additional sources), as well as the date the most recent search was executed.	Page 5
Search	8	Present the full electronic search strategy for at least 1 database, including any limits used, such that it could be repeated.	Page 5
Selection of sources of evidence †	9	State the process for selecting sources of evidence (i.e., screening and eligibility) included in the scoping review.	Page 5
Data charting process ‡	10	Describe the methods of charting data from the included sources of evidence (e.g., calibrated forms or forms that have been tested by the team before their use, and whether data charting was done independently or in duplicate) and any processes for obtaining and confirming data from investigators.	Page 5 & 6
Data items	11	List and define all variables for which data were sought and any assumptions and simplifications made.	Page 5 & 6
Critical appraisal of individual sources of evidence §	12	If done, provide a rationale for conducting a critical appraisal of included sources of evidence; describe the methods used and how this information was used in any data synthesis (if appropriate).	Page 6
Synthesis of results	13	Describe the methods of handling and summarizing the data that were charted.	Page 6
**Results**			
Selection of sources of evidence	14	Give numbers of sources of evidence screened, assessed for eligibility, and included in the review, with reasons for exclusions at each stage, ideally using a flow diagram.	Page 8
Characteristics of sources of evidence	15	For each source of evidence, present characteristics for which data were charted and provide the citations.	Supplementary File S1 (Tables S3–S5)
Critical appraisal within sources of evidence	16	If done, present data on critical appraisal of included sources of evidence (see item 12).	Reported in methods section (Page 6)
Results of individual sources of evidence	17	For each included source of evidence, present the relevant data that were charted that relate to the review questions and objectives.	Page 6–7
Synthesis of results	18	Summarize and/or present the charting results as they relate to the review questions and objectives.	Page 9–13
**Discussion**			
Summary of evidence	19	Summarize the main results (including an overview of concepts, themes, and types of evidence available), link to the review questions and objectives, and consider the relevance to key groups.	Page 13–16
Limitations	20	Discuss the limitations of the scoping review process.	Page 15 & 16
Conclusions	21	Provide a general interpretation of the results with respect to the review questions and objectives, as well as potential implications and/or next steps.	Page 16 & 17
**Funding**			
Funding	22	Describe sources of funding for the included sources of evidence, as well as sources of funding for the scoping review. Describe the role of the funders of the scoping review.	Page 17

JBI = Joanna Briggs Institute; PRISMA-ScR = Preferred Reporting Items for Systematic reviews and Meta-Analyses extension for Scoping Reviews. * Where *sources of evidence* (see second footnote) are compiled from, such as bibliographic databases, social media platforms, and Web sites. † A more inclusive/heterogeneous term used to account for the different types of evidence or data sources (e.g., quantitative and/or qualitative research, expert opinion, and policy documents) that may be eligible in a scoping review as opposed to only studies. This is not to be confused with *information sources* (see first footnote). ‡ The frameworks by Arksey and O’Malley (6) and Levac and colleagues (7) and the JBI guidance (4, 5) refer to the process of data extraction in a scoping review as data charting. § The process of systematically examining research evidence to assess its validity, results, and relevance before using it to inform a decision. This term is used for items 12 and 19 instead of “risk of bias” (which is more applicable to systematic reviews of interventions) to include and acknowledge the various sources of evidence that may be used in a scoping review (e.g., quantitative and/or qualitative research, expert opinion, and policy document).
