# Supplementary material for: Effects of Digital Nature Interventions on Positive Psychological Outcomes: A Scoping Review
Source: Behav Sci (Basel). 2026 Jul 22;16(7):1257. doi: 10.3390/bs16071257 (PMC13405956; doi:10.3390/bs16071257)
Supplement: Supplementary file 1 [file behavsci-16-01257-s001.zip › behavsci-4313907-supplementary.pdf]

Table S1: *Systematic Search Strategy, Boolean Operators, and Article Identification Across Databases*

Database	Access Date	Boolean Code/Command	Filters	Identification of Articles
Anthropic. (2025). Claude (Sonnet 4.6) [Large language model]. https://claude.ai	25th Dec, 2026	First round: Search for all peer-reviewed articles (2020–2025) on digital nature interventions and psychological outcomes. Include virtual nature, VR nature, digital nature exposure, nature apps, digital greenspace, digital blue space, 360° nature videos, and related technologies. Include any psychological outcome. Search broadly and maximize recall. Continue searching with alternative terms until no new eligible articles are found. Exclude duplicates and non-eligible studies. Output only verified links from PubMed, GoogleScholar, ScienceDirect or any other database and report the total number of unique articles found.<br>Second round: Repeat the search using different terms and identify new articles not included in previous results. Maximize recall and report only additional unique studies.<br>Third round: Search again for any remaining eligible articles not identified previously. Use alternative keywords and report only new unique studies until no additional articles are found.	No filter needed	42 results
Google Scholar	Dec 10, 2025 to Dec 15 2025<br><br>Jun 6th and 7th 2026	(intitle:"virtual nature" OR intitle:"immersive nature" OR intitle:"digital nature") (resilience OR optimism OR compassion OR "meaning in life" OR wellbeing)	Date of publication (2020-2025)	161 results
PubMed	Nov 15th, 2025 to Nov 17, 2025<br><br>Jun 5th Jun, 2026	("digital nature" OR "virtual nature" OR "immersive nature" OR "nature-based virtual reality" OR "virtual natural environment" OR "VR nature") AND (resilience OR optimism OR compassion OR empathy OR "positive emotion" OR wellbeing OR "well-being" OR connectedness OR engagement OR "meaning in life" OR "purpose in life")	Publication date (1st Jan, 2020 to 25th Dec. 2025); text availability (full-text); article language (English); species (humans); other (exclude preprints)	60 results
Reference tracking	25th Dec, 2026	Not applicable	Not applicable	6 results
Science Direct	Dec 18th 2025 to Dec 22nd, 2025	("virtual nature" OR "immersive nature") AND (resilience OR	Publication years (2020-2025); article type (review	148 results

	optimism OR compassion OR "meaning in life") AND (wellbeing OR "mental health")	articles, research articles); Language (English); Access type (open access and open archive)

Note: Search dates varied across databases as shown. Anthropic (Claude) was used as an auxiliary AI-assisted search tool to supplement traditional database searching. All searches were limited to 2020–2025.

Table S2: *Excluded Full-Text Articles with Rationale for Exclusion Following Eligibility Assessment*

No.	Citations	Reference	Reason for exclusion
1	Ang et al. (2022)	Ang, W. H. D., Chew, H. S. J., Dong, J., Yi, H., Mahendren, R., & Lau, Y. (2022). Digital training for building resilience: systematic review, meta-analysis, and meta-regression. *Stress and Hedalth*, *38*, 848–869. https://doi.org/https://doi.org/10.1002/smi.3154	Mentions digital training and its effect on resilience, but does not include nature content in the program.
2	Basurrah et al. (2022)	Basurrah, A. A., Baddar, M. A., & Blasi, Z. Di. (2022). Positive psychology interventions as an opportunity in Arab countries to promoting well-being. *Frontiers in Psychology*, *12*, 1–6. https://doi.org/10.3389/fpsyg.2021.793608	Includes positive psychology but does not include digital nature components
3	Bettis et al. (2022)	Bettis, A. H., Burke, T. A., Nesi, J., & Liu, R. T. (2022). Digital technologies for emotion-regulation assessment and intervention: a conceptual review. *Clin Psychol Sci*, *10*(1), 3–26. https://doi.org/10.1177/21677026211011982	Includes digital technologies but does not mentions nature components
4	Brambilla et al. (2023)	Brambilla, E., Stendal, K., Sundling, V., & Calogiuri, G. (2023). O.2.2-3 Virtual nature as an intervention to promote connectedness with and visitation of nature among university students: a randomized trial. *European Journal of Public Health*, *33*(Supplement_1), ckad133.118. https://doi.org/10.1093/eurpub/ckad133.118	Full text not available
5	Cieślik et al. (2020)	Cieślik, B., Mazurek, J., Rutkowski, S., Kiper, P., Turolla, A., & Szczepańska-Gieracha, J. (2020). Virtual reality in psychiatric disorders: a systematic review of reviews. *Complementary Therapies in Medicine*, *52*(March). https://doi.org/10.1016/j.ctim.2020.102480	Mentions virtual reality as an intervention for psychiatric disorder but does not mentions nature components
6	Cricelli et al. (2025)	Cricelli, G., Newbutt, N., Alexander, J., Ellison, C., & Loetscher, T. (2025). Implementation of virtual reality for neurodivergent individuals: perspectives of disability care staff. *Journal of Enabling Technology*, *19*(2), 128–140. https://doi.org/10.1108/JET-09-2024-0062	Includes virtual reality and neurological outcome but does not mentions nature components.
7	Dandil & Kingston (2025)	Dandil, Y., & Kingston, J. (2025). Personalized mobile apps for mental health and well-being in the general population: a systematic review personalized mobile apps for mental health and well-being in the general population: a systematic review. *Journal of Technology in Human Services*, *0*(0), 1–34. https://doi.org/10.1080/15228835.2025.2491347	Includes digital intervention and psychological outcome, but does not include nature components
8	Graf et al. (2020)	Graf, L., Liszio, S., & Masuch, M. (2020). Playing in virtual nature: improving mood of elderly people using VR technology. *Proceedings of Mensch Und Computer 2020*, 155–164. https://doi.org/10.1145/3404983.3405507	Conference proceeding (not published in a peer-reviewed journal).

9	Hartmann et al. (2025)	Hartmann, P., Apaolaza, V., Paredes, M. R., & D'Souza, C. (2025). Virtual nature experiences on Instagram: how greenfluencers' nature posts drive climate action. *International Journal of Advertising*, *44*(4), 620–650. https://doi.org/10.1080/02650487.2024.2447218	Include digital nature and its association to nature connecters; but lacks psychological outcomes
10	Hernandez et al. (2023)	Hernandez, R., Wilund, K., Solai, K., Tamayo, D., Fast, D., Venkatesan, P., Lash, J. P., Lora, C. M., Martinez, L., Alemañy, G. M., Martinez, A., Kwon, S., Romero, D., Browning, M. H. E. M., & Moskowitz, J. T. (2023). Positive psychological intervention delivered using virtual reality in patients on hemodialysis with comorbid depression: protocol and design for the joviality randomized controlled trial. *JMIR Res Protoc 2023*, *12*, 1–13. https://doi.org/10.2196/45100	Includes virtual reality and psychological outcome but does not mentions nature components.
11	Hussien et al. (2025)	Hussien, R. M., Khalil, T., Nashwan, A. J., Al-Najjar, H., & Khedr, M. A. (2025). Compassionate care in nursing: The role of simulation-based compassionate care on nurse's caring behavior, self-efficacy and compassion competency. *Nurse Education in Practice*, *87*(March), 1–11. https://doi.org/10.1016/j.nepr.2025.104470	Mentions the role of digital platform for compassionate care but does not mention nature components
12	Ji et al. (2025)	Ji, B., Batubara, I. M. S., Batten, J., Peng, X., Chen, S., & Ni, Z. (2025). *Digital health interventions targeting psychological health in parents of children with autism spectrum disorder: a scoping review*.	Mentions Digital health for targeting positive psychological outcome but does not mentions nature components
13	Liebherr et al. (2025)	Liebherr, M., Heyder, A., Brailovskaia, J., Burkardt, T. M., Latrouite, C., Montag, C., & Antons, S. (2025). The digital media-use effects (d-MUsE) model: a comprehensive framework for exploring/studying functional and dysfunctional effects on adolescent wellbeing. *Journal of Behavioral Addictions*, *14*(1), 100–113. https://doi.org/10.1556/2006.2025.00007	Mentions digital media for psychological outcome but does not mentions nature components
14	McEwan et al. (2019)	McEwan, K., Richardson, M., Sheffield, D., Ferguson, F. J., & Brindley, P. (2019). A smartphone App for improving mental health through connecting with urban nature. *International Journal of Environtal Research and Public Health*, *16*, 1–15. https://doi.org/10.3390/ijerph16183373	Excluded based on date restriction (pre-2020).
15	Mitchell et al. (2010)	Mitchell, J., Vella-brodrick, D., & Klein, B. (2010). Positive psychology and the internet : A mental health opportunity. *Electronic Journal of Applied Psychology*, *6*(2), 30–41.	Mentions positive psychological intervention for wellbeing but does not mentions digital platform with nature components.
16	Peres et al. (2025)	Peres, B., Noronha, H., Câmara, J., Lopes, D. S., Jorge, J., & Campos, P. (2025). Can Virtual Nature Enhance the Emotional State of Informal Caregivers? In *DIS '25 - Companion Proceedings of the 2025 ACM Designing Interactive Systems Conference: Designing for a Sustainable Ocean* (Vol. 1, Issue 1). Association for Computing Machinery. https://doi.org/10.1145/3715668.3736375	Conference proceeding (not a peer-reviewed journal article).
17	Podkrajšek & Bogataj (2025)	Podkrajšek, A. H., & Bogataj, D. (2025). Enhancing cognitive and emotional health in older adults	Conference proceeding (not a peer-reviewed journal article)

		through nature-based virtual reality: Literature review. *IFAC-PapersOnLine*, *59*(27), 172–177. https://doi.org/10.1016/j.ifacol.2025.12.098	
18	Roberts et al. (2024)	Roberts, C. M., Hulme, K. A., & McCann, N. (2024). Stepping up psychosis: the use of virtual reality in pre-registration mental health nursing education. *Clinical Simulation in Nursing*, *94*, 1–4. https://doi.org/10.1016/j.ecns.2024.101597	Mentions virtual reality as a digital media but not mention the nature component and the study focuses on learning outcome over psychological outcome.
19	Safari (2024)	Safari, H. (2024). Evaluating the efficacy of nature-inspired virtual environments on stress reduction related work. *International Journal of Computational Health and Machine Learning*, *3*(1).	Early version; published later in 2026.
20	Shankar et al. (2025)	Shankar, R., Bundele, A., & Mukhopadhyay, A. (2025). The effectiveness of virtual reality – based mindfulness interventions for managing stress, anxiety, and depression : protocol for a systematic review and meta-analysis of randomized controlled trials. *JMIR Research Protocols*, *14*. https://doi.org/10.2196/68231	Mentions virtual reality based mindfulness interventions and psychological components but does not mention nature components
21	Shehadeh et al. (2025)	Shehadeh, A., Alshboul, O., Taamneh, M. M., Jaradat, A. Q., Alomari, A. H., & Arar, M. (2025). Advanced integration of BIM and VR in the built environment: enhancing sustainability and resilience in urban development. *Heliyon*, *11*(4), e42558. https://doi.org/10.1016/j.heliyon.2025.e42558	Includes digital platform for resilience but does not mentions nature components and psychological components
22	Shobri & Dahlan (2025)	Shobri, N. I. M., & Dahlan, F. M. (2025). Virtual escapes: exploring the effects of nature-based VR environments during academic breaks. *E-Proceeding*, 706–766.	Not a peer-reviewed journal article (institutional repository output).
23	Shrestha et al. (2025)	Shrestha, T., Chi, C. V. Y., Cassarino, M., Foley, S., & Blasi, Z. Di. (2025). Factors influencing the effectiveness of nature-based interventions (NBIs) aimed at improving mental health and wellbeing: an umbrella review. *Environment International*, *196*, 109217. https://doi.org/10.1016/j.envint.2024.109217	Includes real nature as an intervention for improving wellbeing but not digital include digital platform
24	Simbula & Herold (2025)	Simbula, S., & Herold, M. (2025). Exploring the effects of a smartphone app on psychological well- being in university students: a randomized controlled trial. *Journal of Technology in Behavioral Science*. https://doi.org/10.1007/s41347-025-00583-2	Includes smartphone as a digital platform and psychological outcome but does not mentions nature components in the APP.
25	Weisel et al. (2019)	Weisel, K. K., Fuhrmann, L. M., Berking, M., Baumeister, H., Cuijpers, P., & Ebert, D. D. (2019). Standalone smartphone apps for mental health—a systematic review and meta-analysis. *Npj Digital Medicine*, *2*, 1–10. https://doi.org/10.1038/s41746-019-0188-8	Excluded based on date restriction (pre-2020).
26	Wu et al, (2025)	Wu, H., Li, D., Chen, Z., Tang, X., & Wang, G. (2025). Linking forests, coasts, and people: social media insights into sentiment and wellness perceptions in China's nature reserves. *Trees, Forests and People*, *22*, 1–15. https://doi.org/https://doi.org/10.1016/j.tfp.2025.101068	Included digital nature and positive psychological components for specific nature lovers groups.
27	Wyszynski et	Wyszynski, M., Fock, S., & Niehaves, B. (2025). Restoring	Conference proceeding (not a

al. (2025)	capacity at work: evaluating a virtual nature experience for public administration employees. *Fachtagung Rechts- Und Verwaltungsinformatik (RVI 2025)*, *374*(Rvi), 129–140. https://doi.org/10.18420/rvi2025-110		peer-reviewed journal article).

Note. The tables include 27 articles arranged in alphabetical order by first author. Removal of duplicates occurred prior to screening. The final column presents the reasons for exclusion.

Table S3: *Characteristics of Included Primary Studies*

1. Author(s) and Year	2. Country	3. Objectives	4. Research Design & Source of Data	5. Interventions	6. Sample Size	7. Instruments / Measures	8. Major Findings
Ahn et al. (2025)	South Korea	To investigate the effects of immersive VR nature relaxation on mental health and sleep patterns in students.	RCT (Between Subject-3 Arm Design)	VRelax (nature-based VR) vs. progressive muscle relaxation vs. waitlist	N = 45 (41 analyzed)	K-PROMIS (depression/anxiety), perceived stress levels, subjective sleep patterns	Virtual reality relaxation significantly reduced stress and improved subjective sleep patterns, with effects sustained at a 4-week follow-up.
Appel et al. (2020)	Canada	To evaluate the feasibility and benefits of immersive VR for older adults with sensory, mobility, or cognition impairments.	Experimental (Multi-site Non-randomised)	360-degree VR nature videos via HMD	N = 66 (older adults)	Modified STAI, researcher observations, participant ratings, modified MiDAS questionnaire	VR nature was well-tolerated and significantly increased positive affect while decreasing negative emotions such as sadness and worry.
Appel et al. (2022)	Canada	To evaluate VR therapy for managing responsive behaviors in veterans living with dementia	Prospective, longitudinal, non-randomized interventional clinical trial	VR therapy (Targeted sessions during triggers vs. scheduled recreational sessions)	33 participants	CPS, DRS, PAINAD, CHESS, PPSe and qualitative feedback.	VR therapy is feasible for managing responsive behaviors; all participants chose to engage in multiple sessions
Boffi et al. (2022)	Italy	To conduct a visual post-occupancy evaluation of a restorative	Experimental	VR photography of viewpoints in a restorative garden	N = 321	SAM (pleasure and arousal), restoration scale	Virtual exploration showed the garden was perceived as restorative, especially in specifically designed areas. Participants

1. Author(s) and Year	2. Country	3. Objectives	4. Research Design & Source of Data	5. Interventions	6. Sample Size	7. Instruments / Measures	8. Major Findings
		garden using VR photography.					reported mainly positive emotions with mild arousal, and their behavior was mostly calm, contemplative, and nature-focused.
Browning et al., (2020)	United States	To compare short doses of 360-degree nature videos in VR with outdoor nature for mental health support.	Experimental (Between Subjects Design)	6-minute exposure to outdoor forest, VR forest (360-degree video), or indoor control	N = 89	PANAS; PRS; Shimmer GSR+ (SCL); Disgust Sensitivity Scale; EBS	Outdoor nature boosted positive affect, while VR nature preserved it. Both nature conditions were superior to indoor controls and rated as equally restorative.
Browning et al. (2023)	United States	To test if daily exposure to virtual nature reduces anxiety symptoms in college students.	RCT (Between Subjects Design)	360-degree VR nature videos	N = 40 (final analysis)	PSWQ, MASQ (panic), CES-D, Rumination scale, Godin-Shephard physical activity questionnaire, Engagement with Beauty Scale	Daily virtual nature exposure was associated with stronger mental health benefits regarding worry, panic, and rumination.
Brimelow et al., (2020)	Australia	To reduce apathy and improve mood in residential aged care through virtual reality.	Pre-Post / Single-Group/Mixed methods	VR nature (VRelax)	N = 13 (residents)	PEAR (apathy), OERS (emotions), SSQ	Immersive VRn is feasible and well-accepted in residential aged care, showing a significant reduction in apathy and increased engagement, but no significant effect on mood.
Chan et al. (2021)	Singapore	To investigate whether exposure to virtual natural environments can improve emotional well-being and reduce	Experimental (Within Subjects Design)	VR nature walk (forest vs. urban scenes via HMD)	N = 30 (young adults); N = 20 (seniors)	PANAS; Connectedness to Nature Scale (state version); Cardiovascular measures (ECG-derived HR and HRV); simplified self-report scales	Walking in virtual natural environments reduced negative affect and increased nature connection in young adults, and increased positive affect and connection in seniors, with associated physiological

1. Author(s) and Year	2. Country	3. Objectives	4. Research Design & Source of Data	5. Interventions	6. Sample Size	7. Instruments / Measures	8. Major Findings
		stress in young adults and seniors.					relaxation.
Chirico et al. (2023)	Italy	To compare the pro-environmental effectiveness of awe-inspiring versus non-awe-inspiring virtual scenarios featuring either natural or non-natural stimuli.	Experimental (Between Subjects Design)	VR environments (awe-inspiring vs. non-awe-inspiring; nature vs. non-natural)	N = 119	Dispositional Positive Emotion Scale; BIDR (6 subscales; i.e., Impression Management scale; Self-Deceptive Enhancement Scale); INSS; AS; EBS; ERBI	Compared to a control group, exposure to awe-inspiring virtual nature significantly increased socially engaging pro-environmental behavior but not personally engaging behavior. Furthermore, positive affect and disposition toward environmental protection were significantly correlated with this socially engaging behavior.
Clemente et al. (2024)	Italy	To examine whether connectedness mediates the relationship between exposure to two 3D built virtual reality environments (Arctic vs. urban) and vigor among older adults.	Experimental (Between Subjects Design)	3D VR Arctic environment vs. urban environment	N = 53 (age ≥ 65)	DPES (Awe), EPOMS-Brief (vigor-activity), AWE-S (connectedness), IPQ (sense of presence)	Exposure to the 3D Arctic virtual environment was associated with greater connectedness compared to the urban environment. Connectedness significantly mediated the relationship between virtual environment exposure and vigor.
Donald & Egboluche (2023)	Nigeria	To examine connections between self-agency, exposure to virtual nature, and psychological well-	RCT (Between Subjects Design)	Virtual nature images	N = 72	PANAS, SoAS	Exposure to virtual nature significantly improved psychological well-being (positive affect). Self-agency significantly predicted increased well-being.

1. Author(s) and Year	2. Country	3. Objectives	4. Research Design & Source of Data	5. Interventions	6. Sample Size	7. Instruments / Measures	8. Major Findings
		being.					
Fahey (2025)	United Kingdom	To investigate using VR to support adolescent mental health through self-care.	Mixed-Methods	VR applications for adolescent mental health	N = 60 (screening); N = 19 (testing)	BAI, screening, interview	VR has potential to reintegrate adolescents hospitalized for crises; VR environments can trigger real physical and psychological reactions.
Fang et al. (2025)	China	To explore factors and mechanisms of VR environment design on emotional recovery.	Experimental (3 × 2 × 3 Mixed Design)	VR environment with varied motion-control and sound forms	42 college students (21 male; 21 female; aged 22–25)	STAI-S, Presence Questionnaire, GSR, BVP (physiological)	Unnatural motion-control produced the greatest physiological recovery, suggesting presence is not a prerequisite for stress down-regulation.
Finkler et al. (2025)	New Zealand	To investigate the potential of 360 VR nature videos to enhance emotional well-being and nature connectedness.	Pre-Post / Single-Group Design/ Mixed Methods	360-degree VR nature videos (beach, forest, lake, duck pond)	N = 63	Circumplex model of affect; UEQ-S; IPQ; CNS	Positive affect and nature connectedness significantly improved post-viewing. Participants reported high levels of presence and calmness.
Halamov´a et al. (2025)	Slovakia	To test the effect of a VR intervention (EFT-UFB) on unfinished business, compassion, and stress.	RCT (Between Subjects Design)	VR-based Emotion-Focused Therapy for Unfinished Business (EFT-UFB)	N = 52 (final analysis)	UFB-RS, SOCS, s-SIB, FSCRS, PSS-10	The study did not find robust, statistically significant group differences over time for self-compassion or stress outcomes.
J. Kim et al., (2025)	United States	To examine preliminary efficacy of immersive VR meditation in reducing psychological distress among	Pre-Post / Single-Group Design)	Immersive VR meditation	19 university students	Perceived Stress Scale (PSS-10); State-Trait Anxiety Inventory (STAI - Form Y)	Participation in the immersive virtual reality meditation Intervention program was associated with significant reductions in perceived stress and state anxiety, with

1. Author(s) and Year	2. Country	3. Objectives	4. Research Design & Source of Data	5. Interventions	6. Sample Size	7. Instruments / Measures	8. Major Findings
		university students.					large effect sizes indicating improved psychological well-being following the intervention.
Jones & Wheat (2023)	United Kingdom	To investigate affective and cerebral hemodynamic effects of presence in virtual environments during exercise.	RCT (Within Subjects Design; Repeated Measure)	VR nature vs. 360-degree video vs. control condition	N = 12	Feeling Scale (FS), Attentional Focus scale, PQ, SSQ	Immersive VR promoted a greater sense of presence than 360-degree video. At similar exercise intensities, the 360-degree video was perceived as the most distracting, pleasant, and enjoyable condition, while the control condition showed the greatest prefrontal brain activation.
B. Kim et al. (2025)	South Korea	To examine VR-based treatment for individuals with depression using mindfulness cognitive therapy.	Pre-Post / Single-Group Design	VR-MBCT program: 4 sessions (mindfulness, starry expressions, self as context, acceptance)	N = 73 (38 with depression, 35 without)	SUS; IPQ; NASA-TLX; EDA (entropy analysis); Eye-tracking	VR-based mindfulness cognitive therapy incorporating virtual natural environments was feasible for individuals with depression and showed favorable emotional responses.
Kluge et al. (2023)	Australia	To evaluate a VR platform (Performance Edge) for stress management training in a defense workforce.	Pre-Post / Single-Group/Mixed methods	VR stress management training ("Performance Edge")	N = 189	Presence Questionnaire, User Engagement Scale, State Mindfulness Scale, Relaxation Inventory, respiratory rate	Trainees reported high levels of user engagement, immersion, and privacy. The ability to practice cognitive strategies in diverse environments, especially in group settings, emerged as highly valuable.
Kumpulainen et al. (2025)	Finland	To investigate the psychophysiological benefits of a nature-based soundscape	RCT (Within Subjects Design; Crossover)	Nature-based soundscape (nature sounds with music elements)	N = 53 (37 women, 16 men)	HRV; Warr's Affective Well-Being Questionnaire, VAS	Compared to city soundscapes, nature-based soundscapes significantly enhanced physiological relaxation, comfort, enthusiasm, creativity, and sense

1. Author(s) and Year	2. Country	3. Objectives	4. Research Design & Source of Data	5. Interventions	6. Sample Size	7. Instruments / Measures	8. Major Findings
		compared with a reference soundscape and examine effects on heart rate variability (HRV), physiological responses, affective well-being, creativity, and belonging.					of belonging while reducing anxiety- and depression-related affect, suggesting they promote immediate psychophysiological recovery in healthy adults.
Lee et al. (2025)	South Korea	To explore types of digital therapeutic gardening and their relationship with mental health outcomes.	Survey / Cross-Sectional Design	Digital therapeutic gardens (media-based, nature-based, app-based, smart gardens)	N = 335 (community-dwelling adults)	Mental Health Screening Tool for Depression-2; Mental Health Screening Tool for Anxiety-2; Korean PSS-10; Core Life Activities Scale; SWLS; UCLA Loneliness Scale-8; Lubben Social Network Scale-6	Participants with digital therapeutic garden experience reported greater life satisfaction, vitality, and stronger social networks. Garden experience was associated with lower stress among those with higher depression or anxiety.
Lehto et al. (2025)	United States	To evaluate accessibility and preliminary emotional health and quality of life outcomes of hospice caregivers through a nature-based VR intervention.	Pre-Post / Single-Group	Nature-based VR intervention	N = 15	AIM, FIM, VR sickness questionnaire; The PROMIS-29	Significant improvements were observed in overall quality of life and a reduction in anxiety symptoms, with minimal VR sickness.
Litleska	Norway	To evaluate	RCT	360-degree	N = 60	PRS, SSQ,	Virtual green

1. Author(s) and Year	2. Country	3. Objectives	4. Research Design & Source of Data	5. Interventions	6. Sample Size	7. Instruments / Measures	8. Major Findings
re et al. (2022)		the impact of different VR modes (360-degree video vs. 3D model) during green exercise.	(Between Subjects Design)	VR nature video vs. 3D VR nature model during treadmill walking	(healthy adults)	PAAS (affect), blood pressure kit, RPE, enjoyment, LTEQ, CNS, HR-monitor	exercise elicited higher enjoyment than walking alone. 3D models provided a stronger sense of presence than 360-degree videos.
Lundstedt et al. (2021)	Sweden	To investigate how to design virtual natural environments (VNEs) for older adults in residential care.	Qualitative (Interview/ Observation)	Virtual natural environments (VNEs) (e.g., "theBlu", "VR Island")	N = 7 (residents)	Observational notes, open-ended questions, staff interviews	Awe-inspiring virtual natural environments invoked the most fascination and joy, suggesting VR nature can improve short-term mood in residential care.
Marocco et al. (2025)	Italy	To explore restorative effects of preferred virtual natural environments on emotions and physiological responses.	Experimental (Mixed Design)	Preferred VR natural environments (forest, meadow, tropical island)	N = 52	Screening Questions; SAM scale; Polar H10	Preferred natural environments enhanced positive emotional valence and reduced arousal following stress. Both virtual natural and neutral settings reduced arousal.
Matsangidou et al. (2025)	Cyprus	To examine the influence of VR on behavioral and psychological symptoms of dementia (BPSD).	Mixed-Methods	VR system with 10 selectable environments	N = 20	MMSE, Heart Rate, Eye-tracking, semi-structured interviews	VR significantly reduced behavioral and psychological symptoms (aggression, anxiety, apathy, depression) associated with dementia and improved quality of life.
McEwan et al. (2020)	United Kingdom	To evaluate a smartphone app-based wellbeing interventio	RCT (Between Subjects Design)	"Shmapped" smartphone-based wellbeing intervention	N = 228 (completed post-intervention measures)	Thematic Analysis	A smartphone app-based digital nature intervention (urban nature engagement) improved wellbeing and nature connectedness, with

1. Author(s) and Year	2. Country	3. Objectives	4. Research Design & Source of Data	5. Interventions	6. Sample Size	7. Instruments / Measures	8. Major Findings
		n designed to improve mental health by prompting users to notice the good things about urban nature.					qualitative themes reflecting positive emotional responses to nature.
McEwan et al. (2022)	United Kingdom	To assess the feasibility and impact of online forest bathing for adults with Long-COVID.	Pre-Post / Single-Group (Repeated Measure)	Online forest bathing sessions	N = 22 (waitlist); N = 16 (post-intervention)	DPES (Awe), EPOMS-Brief (vigor-activity), AWE-S (connectedness), IPQ (sense of presence)	Compared with the waitlist control, online forest bathing significantly reduced anxiety, rumination, and Long-COVID symptoms, while increasing social connection and overall well-being.
Mostajeran, Fischer, et al. (2023)	Germany	To study the effects of immersive computer-generated nature on affect and cognition.	Experimental (Within Subjects Design)	CG virtual nature vs. neutral environment	N = 27	TMT, DSM, PRS, PANAS, PSS, IPQ, SSQ	Virtual nature exposure significantly improved executive functioning and memory, while enhancing perceived restorativeness and positive affect, and reducing stress.
Mostajeran, Steinicke, et al. (2023)	Germany	To determine if adding virtual plants leads to higher cognitive performance and well-being in VR.	Experimental (Within Subjects Design)	Addition of virtual plants to VR environment	N = 39	PANAS, ZIPERS, Digit Span Backward, PRS, Sense of Presence	Adding virtual plants led to significantly higher positive affect, attentive coping, perceived restorativeness, and sense of presence.
Ochiai et al. (2023)	Japan	To examine the physiological and psychological relaxation effects of nature sound	Experimental (Within Subjects Design; Crossover)	Nature sounds vs. city sounds (auditory intervention)	N = 22	NIRS (prefrontal cortex oxy-Hb), HRV, Modified SD method, POMS2	Nature sounds significantly reduced bilateral prefrontal cortex oxy-Hb levels, indicating physiological relaxation. Negative mood and total mood disturbance decreased, while

1. Author(s) and Year	2. Country	3. Objectives	4. Research Design & Source of Data	5. Interventions	6. Sample Size	7. Instruments / Measures	8. Major Findings
		exposure on patients with gambling disorder.					positive mood, comfort, relaxation, and feelings of naturalness increased. No significant changes were observed in HRV.
Oe et al. (2025)	Japan	To explore relationships between digital forest bathing and well-being, and to compare male and female responses.	Survey / Cross-Sectional (Qualitative Comparative)	Digital forest bathing (DX)	N = 347 (Female: 197; Male: 150)	Structured surveys	Positive evaluation of forest bathing significantly predicted perceived stress reduction. Females showed skepticism despite a predisposition to traditional forest immersion, whereas males showed greater receptivity to digital alternatives.
Ojala et al. (2022)	Finland	To determine if short virtual nature breaks in the office can reduce stress.	Experimental (Repeated Measure)	Virtual nature room (video + audio vs. sound-only vs. silence)	N = 39	ROS, PANAS, SVS, Anxiety measure, HRV	Forest and water videos with sounds promoted the best recovery through increased restorativeness and decreased heart rate.
Owens & Bunce (2023)	United Kingdom	To test the effect of brief exposure to a virtual woodland walk on adolescent mental wellbeing.	Experimental (Proof-of-Principle)	6-minute video of virtual woodland walk vs. urban train journey (audio-visual)	N = 76 (adolescents)	SWEMWBS; I-PANAS-SF; Brief State Rumination Index; PSS-4; Nature Connection Index; bespoke nature spirituality items	The virtual nature condition improved stress, relaxation, mood, and attention, while significantly increasing nature spirituality and connection.
Reese et al., (2022)	Germany	To compare virtual nature experiences with physical forest bathing for stress and well-being.	Experimental (Between Subjects Design)	VR forest walk vs. physical forest walk	N = 50	PANAS, SSS (stress), SVS (vitality), ROS, PRS-11	Both virtual and physical forest walks significantly increased positive affect and decreased negative affect. Physical walks were superior only for subjective vitality.
Smalley	United	To explore	RCT	Virtual	N = 8,752	Perceived	Natural sounds were

1. Author(s) and Year	2. Country	3. Objectives	4. Research Design & Source of Data	5. Interventions	6. Sample Size	7. Instruments / Measures	8. Major Findings
et al. (2023)	Kingdom	factors (soundscapes, music, and memories) influencing emotional responses to virtual nature.	(Between Subjects Design)	nature visuals with varied acoustic soundtracks	(7,636 final)	restorative potential (adapted), Calmness, Excitement, Awe, Nostalgia	more restorative and calming than music. Positive memories were strongly associated with higher perceived restoration.
Spangenberger et al. (2025)	Germany	To investigate how virtual body ownership (embodying a tree) influences nature connectedness and affective processes.	Experimental (Between Subjects Design)	VR "Tree" application (tree growth experience with synchronous branch movement)	N = 85 (43 non-threatening, 42 threatening)	Inclusion of Nature in Self scale; Virtual Body Ownership scale; PANAS; Compassion items; IPQ; VRSQ; Electrodermal activity	Embodying nature in VR significantly increased nature connectedness. Compassion mediated this relationship, and threatening scenarios significantly increased compassion, anger, and anxiety.
Szitás et al. (2025)	Slovakia	To explore the effects of a virtual forest vs. a virtual urban setting on self-compassion and stress.	RCT (Within/Between Subjects)	3D VR models of forest vs. city environment (360-degree view)	N = 28	SCCS; PSS-10; s-SIB; SOCS; FSCRS; Nature Exposure Scale; Igroup Presence Questionnaire	The forest group showed increased state self-compassion and decreased self-criticism. The city group experienced increased perceived stress and declined trait compassion.
Theodorou et al. (2023)	Italy	To investigate if different virtual environments enhance subjective vitality through restorativeness.	RCT (Between Subjects Design)	360-degree panoramic photos (urban, park, lake, arctic) via HMD	N = 113 (university students)	PRS-11; Subjective Vitality Scale; PSS; MSAQ; IPQ	Virtual natural environments significantly enhanced restorativeness, which in turn increased feelings of subjective vitality compared to urban settings.
van Houwelingen-snippe, van Rompay, & Allouch	Northern Europe, Canada, United States	To investigate if digital nature increases community connectedness and	Experimental (Between Subjects Design)	Walkthrough videos of digital nature (wild vs. tended; spacious vs. dense)	N = 1,203	Inclusion of Community in the Self Scale; Social Aspiration Scale; Awe Experience Scale;	Community connectedness increased after viewing. Tended nature elicited more social aspirations than wild nature. Distance from nature

1. Author(s) and Year	2. Country	3. Objectives	4. Research Design & Source of Data	5. Interventions	6. Sample Size	7. Instruments / Measures	8. Major Findings
(2020)		explore loneliness predictors.		with bird and footstep sounds		Perceived Restorativeness Scale; UCLA Loneliness Scale; Spatial Presence Experience Scale; Nature Relatedness Scale	predicted higher loneliness.
van Houwelingen-snippe, van Rompay, de Jong, et al. (2020)	The Netherlands	To explore if digital nature projections stimulate social aspirations, related emotions, and the role of social context.	Experimental (Mixed Design)	Digital nature projections (32x9 immersive format) depicting wild or tended nature with bird sounds and forest scents	N = 96	Social Aspirations Scale; Awe Experience Scale; Perceived Restorativeness Scale; Spatial Presence Experience Scale	Spacious scenes elicited significantly higher social aspiration and awe scores, particularly when watching alone. Dense nature was rated more fascinating than spacious nature in social viewing conditions
Veling et al. (2021)	The Netherlands	To compare virtual reality relaxation (VRelax) with standard relaxation exercises in psychiatric outpatients.	RCT (Within Subjects Design; Crossover)	VRelax app (360-degree nature videos via HMD) vs. audio-based standard relaxation	N = 50 (49 analyzed)	VAS (momentary affect), PSS, IDS-SR, BAI, GPTS, SSQ	VRelax, using immersive nature-based environments, significantly reduced negative affect and improved positive affect immediately after use, with stronger effects than standard relaxation exercises, while longer-term effects on anxiety and mood outcomes were modest.
Wang et al. (2020)	Taiwan	To explore psychological and physiological responses in GAD patients using exercise and VR.	RCT (Between Subjects Design)	VR nature landscape vs. abstract paintings during exercise	N = 77	GAD-7, Perceived Stress Scale, EEG (alpha waves)	The virtual nature group demonstrated significantly higher alpha wave values (relaxation) and lower perceived stress after exercise.
Wong et al. (2025)	Hong Kong, China	To explore therapeutic mechanism	Qualitative (Interview/ Thematic	VR-based stress reduction	N = 35	Semi-structured interviews;	Participants experienced relaxation and

1. Author(s) and Year	2. Country	3. Objectives	4. Research Design & Source of Data	5. Interventions	6. Sample Size	7. Instruments / Measures	8. Major Findings
		s of virtual nature for young adults' mental well-being.	Analysis)	intervention		thematic analysis	calmness. A model revealed bidirectional relationships between sensory elements and emotional impacts.
Yeo et al. (2020)	United Kingdom	To explore if three modes of virtual nature reduce negative affect and/or increase positive affect.	RCT (3 Levels between Subjects Design)	Underwater coral reef viewed via 2D TV, 360-degree VR video, or interactive computer-generated VR (CG-VR)	N = 96	Presence and Reality Judgement Questionnaire; MSBS (boredom); SPANE (mood); INS (nature connectedness)	All conditions reduced boredom and negative affect. Interactive CG-VR was significantly more effective than 2D TV at increasing positive affect and nature connection.
Zeng et al. (2025)	China	To examine cancer patients' acceptance of VR-based interventions for emotional self-regulation by identifying factors influencing their behavioral intention to use VR.	Survey / Cross-Sectional	VR technology intervention for emotional regulation	N = 489 (cancer patients)	TAM-based survey; various scales	In cancer patients, exposure to digital nature is associated with positive psychological outcomes. Perceived usefulness, immersion, subjective norms, and personal innovativeness are positively associated with adaptive responses, whereas technological anxiety and perceived risk are negatively associated.

Note. The comprehensive table includes 46 primary articles arranged in alphabetical order by first author. The columns include author(s) and year, country, objectives, research design and source of data, interventions, sample size, instruments and measures, and major findings.

Table S4: *Characteristics of Included Review Articles*

1. Author(s) and Year	2. Objectives	3. Source Type / Design	4. Intervention / Concept Included in the Reviews	5. Sample Size	6. Framework, Quality Assessment & Instruments / Measures	7. Major Findings
Abdullah et al.,	To conduct a systematic	Systematic qualitative	Virtual nature/forest	18 articles	PRISMA guidelines;	The study suggests that virtual reality

1. Author(s) and Year	2. Objectives	3. Source Type / Design	4. Intervention / Concept Included in the Reviews	5. Sample Size	6. Framework, Quality Assessment & Instruments / Measures	7. Major Findings
(2021)	qualitative review on the impact of virtual nature therapy on stress responses.	review	therapy (visual, auditory, and olfactory stimuli)		validated psychometric and physiological measures (including POMS, PANAS, VAS, SSS, PSS, STAI-S, HR, HRV, SBP, DBP, SCL)	(VR) exposure to nature can reduce stress and promote relaxation, showing beneficial effects comparable to real natural environments, although further research is needed to confirm its effectiveness.
Brambilla et al. (2024)	To systematically review and synthesize evidence on the effects of immersive virtual nature (IVN) on nature connectedness.	Systematic review and meta-analysis	Immersive Virtual Nature (IVN) vs. non-immersive virtual nature, immersive/non-immersive virtual built environments, and actual nature	9 studies (total N = 730 participants)	PRISMA guidelines; PICOS framework; validated scales including CNS-State, CNS-Trait, INS, NRS, SINS	Immersive virtual nature experiences were associated with increased nature connectedness, offering mental well-being benefits when access to real nature is limited.
Chen et al. (2025)	To evaluate the impact of virtual natural environments on stress, anxiety, and depression in healthy adults.	Systematic review and meta-analysis	Virtual natural environments (2D, 360-degree, and VR head-mounted displays)	24 studies (total N = 1,471 for anxiety outcomes)	PRISMA guidelines; PICOS framework; validated psychometric scales (including BDI-II, HADS, STAI, DASS, POMS, VAS)	Virtual nature exposure effectively reduces anxiety (large effect), stress, and depression (moderate effects). Brief exposure (10–15 minutes) is recommended for anxiety relief.
Corbel et al. (2025)	To scope the effects of VR on pain and anxiety in older adults.	Scoping review	VR (passive nature and active games)	17 studies	PRISMA-ScR guidelines; Delphi list for quality assessment	VR interventions show potential for pain management and anxiety reduction in older adults, though results vary by content type. VR effectively reduced anxiety, but effects on pain were promising yet

1. Author(s) and Year	2. Objectives	3. Source Type / Design	4. Intervention / Concept Included in the Reviews	5. Sample Size	6. Framework, Quality Assessment & Instruments / Measures	7. Major Findings
						occasionally non-significant.
Fan & Baharum (2024)	To assess the effects of digital nature vs. actual nature on stress reduction.	Systematic review and meta-analysis	Digital nature (static images, 360-degree videos) compared to real-world natural environments	10 articles (total N = 886 participants)	PRISMA guidelines; Review Manager (RevMan) for meta-analysis	Digital natural environments provide the same level of stress recovery as actual nature when intervention content is consistent.
Frost et al. (2022)	To systematically review the psychological effects of virtual immersion in nature on human well-being.	Systematic review (quantitative studies)	VR-based immersion in nature	21 studies (total N = 1,301 participants)	PRISMA guidelines; PICO framework; GRADE system for quality assessment	Evidence suggests virtual nature significantly decreases negative affect; however, results for positive affect and physiological stress responses were inconsistent.
Hubbard et al. (2025)	To report the effects of virtual reality nature (VRn) on the mental health of higher education students.	Systematic review and meta-analysis	Virtual reality nature (CGI or 360-degree videos)	24 articles (total N = 1,419 participants)	Modified Downs and Black checklist; validated psychometric scales assessing mood, anxiety, stress, and cognition	Most studies reported beneficial pre-post effects of virtual reality nature on mood, anxiety, stress, and cognition. No significant differences were found between VR nature and flat-screen nature for anxiety.
Li et al. (2021)	To address benefits derived from virtual nature and compare VR to traditional media.	Mini-review	VR nature (360-VR and CG-VR) delivered via HMDs	Not applicable	Not applicable	Virtual nature induces relaxation, restores attention, and improves pain experiences. Interactive CG-VR may provide a greater sense of presence than 360-degree videos.
Lin et al. (2025)	To theorize how and why VR leads to human well-being through	Scoping review	VR elements (environment, interaction, virtual body) inducing illusions (Place, Plausibility, and	187 articles coded	PRISMA-ScR guidelines; inductive coding; validated well-being scales	Place Illusion primarily enhances subjective well-being (restoration/awe). Plausibility and Body Ownership illusions link more strongly to

1. Author(s) and Year	2. Objectives	3. Source Type / Design	4. Intervention / Concept Included in the Reviews	5. Sample Size	6. Framework, Quality Assessment & Instruments / Measures	7. Major Findings
	design and mechanisms.		Body Ownership)			psychological well-being.
Lopes & Falk (2024)	To investigate multisensory digital nature setups and their outcomes for stress and anxiety reduction.	Systematic review	Multisensory immersive VR (audio-visual-olfactory, sometimes including tactile/somatosensory stimuli)	14 articles	PRISMA guidelines; NIH-NHLBI quality assessment tool; modified JBI checklists	Including olfactory stimuli yields positive effects comparable to conventional natural environments. Multisensory digital nature holds promise for mental health interventions.
Ma et al. (2023)	To investigate if VR-based mindfulness training can improve mental health outcomes in adults.	Systematic review	Immersive VR-based mindfulness training	7 studies (total N = 798 participants)	SIGN 50 Checklist; CRD guidelines	VR-assisted mindfulness is more effective than conventional mindfulness for state mindfulness, anxiety, depression, and sleep quality.
Monaghesh et al. (2022)	To review virtual reality-based interventions for patients with paranoia.	Systematic review	VR-based therapeutic interventions (HMD and Unity3D)	8 studies	CASP Randomized Controlled Trial Standard Checklist	VR interventions are effective for improving social participation, reducing anxiety, and diminishing paranoid symptoms.
Restout et al. (2023)	To assess the effectiveness of 360 VR videos on well-being in older adults with or without cognitive impairment	Scoping review	Fully immersive VR interventions through 360-degree videos	10 articles (524 total participants)	PRISMA-SR; various scales (e.g., PANAS, PEAR, GDS, STAI)	VR 360 videos are feasible, safe, and ensuring enjoyment. They show beneficial effects on emotions, apathy, and social engagement in the elderly
Saboor et al. (2024)	To evaluate the effectiveness of digital positive psychology interventions (PPIs) for well-being in young	Systematic review and meta-analysis	Web-based, digital, or smartphone-based interventions using a positive psychology framework	35 articles (total N = 7,341 participants)	PRISMA guidelines; CONSORT checklist	Digital positive psychology interventions significantly improve purpose, gratitude, and hope (medium-to-large effect). They also enhance compassion and coping while

1. Author(s) and Year	2. Objectives	3. Source Type / Design	4. Intervention / Concept Included in the Reviews	5. Sample Size	6. Framework, Quality Assessment & Instruments / Measures	7. Major Findings
	people.					reducing stress.
Sadowski & Khoury (2022)	To review nature-based mindfulness-compassion programs using VR for older adults.	Narrative review	Nature-based mindfulness-compassion programs using VR	6 reviews/meta-analyses; 23 empirical studies (8 nature-based VR in older adults, 8 mindfulness-based VR, 7 compassion-based VR)	Not specified	VR integrating nature, mindfulness, and compassion provides accessible, safe, and engaging alternative mental health care for older adults. However, rigorously designed empirical studies are required to validate these interventions and address the critical needs of the rapidly aging global population.
Spano et al. (2023)	To systematically review the psychological and psychophysiological benefits of virtual nature.	Systematic review	Virtual nature exposure (green/blue spaces) via HMDs or 2D screens	59 articles	PRISMA guidelines; 8-item quality assessment checklist	Virtual nature exerts a positive effect on users, with major outcomes including mood improvement, stress reduction, and enhanced restorativeness.
Van Houwelingen-Snippe et al. (2021)	To assess the current understanding and intervention strategies for improving well-being among older adults through virtual reality representations of nature.	Rapid review	Not specified	27 articles (3 articles for Nature based intervention)	Not specified	Evidence on digital nature interventions for improving well-being among older adults was limited, and further research was recommended to understand the effects of virtual nature characteristics and the underlying psychological processes.
Wen et al. (2024)	To comprehensively assess virtual nature immersion for patient	Systematic review and meta-analysis	Immersive VR natural environments (blue/green spaces)	30 trials (total N = 2,123 participants)	PRISMA guidelines; Extended PICO framework; validated psychometric	Virtual nature significantly improved pain, anxiety, and fear in patients. Natural scenes also lowered heart rate and

1. Author(s) and Year	2. Objectives	3. Source Type / Design	4. Intervention / Concept Included in the Reviews	5. Sample Size	6. Framework, Quality Assessment & Instruments / Measures	7. Major Findings
	well-being, physiological health, and recovery.				and physiological measures (including VAS, NRS, HADS, STAI, heart rate, DBP, SpO2)	diastolic blood pressure.
Wiebe et al. (2022)	To review virtual reality in the diagnosis and therapy of mental disorders.	Systematic review	VRET, VCET, various VR interventions	721 articles	Risk of Bias assessment using Yes/No criteria based on PRISMA guidelines	VRET demonstrates high effectiveness for paranoia and anxiety therapy. VR assessment accurately distinguishes dementia from healthy states. Evidence supports VRET for anxiety, PTSD, addiction disorders, cognitive training in dementia, and social skill training in autism spectrum disorder.

Note: The comprehensive table includes 17 review articles arranged in alphabetical order by first author. The columns include author(s) and year, objectives, source type and design, intervention or concept included in the reviews, sample size, framework and quality assessment and instruments or measures, and major findings.

Table 5: *Characteristics of Included Conceptual and Theoretical Papers*

1. Author(s) and Year	2. Objectives	3. Source Type / Design	4. Concept	5. Major Findings
Fuentes et al. (2024)	To conceptualize digital placemaking in nature for well-being.	Conceptual / Theoretical Review	Digital placemaking in nature	Proposes a model to reframe human-nature relationships in cities through digital extensions of physical space for well-being.
Williams & Langley (2021)	To explore perspectives and prospects for immersive virtual nature art and well-being.	Conceptual / Theoretical review	Immersive virtual nature art	Virtual nature art shows potential to support well-being through restorative immersive experiences based on biophilic design.

Note. The comprehensive table includes 2 conceptual articles arranged in alphabetical order by first author. The columns include author(s) and year, objectives, source type and design, concept, and major findings.

List of the Abbreviations (Alphabetical Order)

1. 360-VR: 360-degree Virtual Reality
2. AIM: Acceptability of Intervention Measure

3. AWE-S: Awe Experience Scale
4. BAI: Beck Anxiety Inventory
5. BDI-II: Beck Depression Inventory-II
6. BVP: Blood-Volume Pulse
7. CASP: Critical Appraisal Skills Programme
8. CES-D: Center for Epidemiological Studies Depression Scale
9. CGI: Computer-Generated Imagery
10. CHESS: Comprehensive Health Enhancement Support System
11. CNS: Connectedness to Nature Scale
12. CONSORT: Consolidated Standards of Reporting Trials
13. CPS: Chronic Pain Scale
14. CRD: Centre for Reviews and Dissemination
15. DASS: Depression Anxiety Stress Scales
16. DBP: Diastolic Blood Pressure
17. DX: Digital transformation
18. EBS: Engagement with Beauty Scale
19. ECG: Electrocardiogram
20. EDA: Electrodermal Activity
21. EEG: Electroencephalogram
22. FIM: Feasibility of Intervention Measure
23. FS: Feeling Scale
24. FSCRS: Forms of Self-Criticizing/Attacking and Self-Reassuring Scale
25. GAD-7: Generalized Anxiety Disorder-7
26. GDS: Geriatric Depression Scale
27. GPTS: Green et al. Paranoid Thought Scales
28. GRADE: Grading of Recommendations Assessment, Development and Evaluation
29. GSR: Galvanic Skin Response
30. HADS: Hospital Anxiety and Depression Scale
31. HR: Heart Rate
32. HRV: Heart Rate Variability
33. I-PANAS-SF: International Positive and Negative Affect Schedule Short Form
34. IDS-SR: Inventory of Depressive Symptomatology — Self-Report
35. INS: Inclusion of Nature in Self Scale
36. IPQ: Igroup Presence Questionnaire
37. JBI: Joanna Briggs Institute (Critical Appraisal Checklists)
38. K-PROMIS: Korean-Patient-Reported Outcomes Measurement Information System
39. LTEQ: Leisure Time Exercise Questionnaire
40. MASQ: Mood and Anxiety Symptom Questionnaire
41. MBCT: Mindfulness-Based Cognitive Therapy
42. MiDAS: Music in Dementia Assessment Scales
43. MMSE: Mini-Mental State Examination
44. MSAQ: Motion Sickness Assessment Questionnaire
45. MSBS: Multidimensional State Boredom Scale
46. NASA-TLX: NASA-Task Load Index
47. NIH-NHLBI: NIH National Heart, Lung, and Blood Institute Quality Assessment Tool
48. NRS: Numerical Rating Scale
49. OERS: Observational Emotional Rating Scale
50. PAAS: Physical Activity Affect Scale
51. PAINAD: Pain Assessment in Advanced Dementia
52. PANAS: Positive and Negative Affect Schedule
53. PEAR: Person-Environment Apathy Rating
54. PICO: Population, Intervention, Comparison, Outcome
55. PICOS: Population, Intervention, Comparison, Outcome, Study Design
56. POMS: Profile of Mood States
57. PPIs: Positive Psychology Interventions
58. PPSe: Psychoticism, Psychopathy, and Sadism scale
59. PQ: Presence Questionnaire

60. PRISMA-SR: Preferred Reporting Items for Systematic Reviews and Meta-Analyses (used for systematic reviews)
61. PROMIS-29: Patient-Reported Outcomes Measurement Information System (29 items)
62. PRS: Perceived Restorativeness Scale
63. PSS: Perceived Stress Scale
64. PSWQ: Penn State Worry Questionnaire
65. ROS: Restorative Outcome Scale
66. RPE: Rating of Perceived Exertion
67. RS: Resilience Scale
68. SAM: Self-Assessment Manikin
69. SBP: Systolic Blood Pressure
70. SCCS: Self-Compassion and Self-Criticism Scales
71. SCL: Skin Conductivity Level
72. SD: Semantic Differential method
73. SIGN 50: SIGN 50 Checklist
74. SINS: State Integration of Nature in Self scale
75. SoAS: Sense of Agency Scale
76. SOCS: Sussex-Oxford Compassion Scale
77. s-SIB: short Scale for Interpersonal Behaviour
78. SPANE: Summary of Positive and Negative Experience
79. SSQ: Simulator Sickness Questionnaire
80. SSS: Standard Stress Scale
81. STAI: State-Trait Anxiety Inventory
82. STAI-S: State-Trait Anxiety Inventory-State
83. SUS: System Usability Scale
84. SVS: Subjective Vitality Scale
85. SWEMWBS: Short Warwick-Edinburgh Mental Well-being Scale
86. SWLS: Satisfaction With Life Scale
87. TAM: Technology Acceptance Model
88. TMT: Trail Making Test
89. UEQ-S: User Experience Questionnaire-Short
90. UFB-RS: Unfinished Business Resolution Scale
91. VAS: Visual Analogue Scale
92. VNEs: Virtual Natural Environments
93. VR-MBCT: Virtual Reality-Mindfulness-Based Cognitive Therapy
94. VRET: Virtual Reality Exposure Therapy
95. VRn: Virtual Reality nature
96. VRSQ: Virtual Reality Sickness Questionnaire
97. ZIPERS: Zuckerman inventory of personal reactions

Note: These 97 abbreviations are solely from the data extraction tables (3-5). The main body of the article does not include all these abbreviations.
